# A systematic review of implant materials for facial reconstructive and aesthetic surgery

**DOI:** 10.3389/fsurg.2025.1548597

**Published:** 2025-03-28

**Authors:** Martin Kauke-Navarro, Leonard Knoedler, Helena Baecher, Khalil Sherwani, Samuel Knoedler, Omar Allam, Fortunay Diatta, Michael Alperovich, Ali-Farid Safi

**Affiliations:** ^1^ Department of Surgery, Division of Plastic Surgery, Yale School of Medicine, New Haven, CT, United States; ^2^ Department of Oral and Maxillofacial Surgery, Charité—Universitätsmedizin Berlin, Corporate Member of Freie Universität Berlin, Humboldt-Universität zu Berlin, and Berlin Institute of Health, Berlin, Germany; ^3^ Department of Cranio- and Maxillofacial Surgery, University Hospital Regensburg, Regensburg, Germany; ^4^ Faculty of Medicine, University of Vienna, Vienna, Austria; ^5^ Faculty of Medicine, University of Bern, Bern, Switzerland

**Keywords:** facial implants, facial implantology, face design, facial reconstruction, aesthetic facial surgery, craniomics

## Abstract

**Background:**

Assessing facial plastic surgery techniques is essential for improving patient safety and outcomes through evidence-based practices. Despite the extensive use of facial implants, there is a scarcity of thorough research on their long-term effects and safety profiles.

**Methods:**

A systematic review was conducted following PRISMA guidelines, analyzing studies from 1970 to 2024 on various implant materials for facial reconstruction and augmentation. The databases searched for this review included PubMed, Web of Science, Google Scholar, and EMBASE. Inclusion criteria were full-text articles in English, focusing on alloplastic materials for craniofacial skeleton replacement or augmentation.

**Results:**

We included 117 studies with 4,273 patients and a mean follow-up of 34 months (range: 15 days to 25 years). Of these, 56% focused on reconstruction, 33% on aesthetics, and 10% on both. Patient ages ranged from 6 months to 85 years, with most studies addressing the orbital (29%), chin (22%), and malar (19%) regions. 67% of studies evaluated potential complications and found an overall rate of 4.4%. Nerve injuries (2.1%) and infections (1.0%) were the most frequent issues, with hematoma, implant displacement, and bone resorption rates at 1.4%, 0.59%, and 0.68%, respectively. Patient-specific implants (PSIs) showed promise in reducing complications such as infections, suggesting that customization to patient anatomy may provide benefits. The highest rate of complication-free postoperative recovery was observed with polyethylene facial implants.

**Conclusion:**

This review highlights variability in implant performance. The increased use of PSI suggests improved outcomes, warranting further investigation. Standardized outcome reporting and further research are needed to enhance comparability and guide clinical practice.

**Systematic Review Registration:**

PROSPERO, identifier (CRD42024501754).

## Introduction

Alloplastic facial implants are routinely used to correct facial asymmetries, defects, and deformities. Esthetic balancing surgeries are increasingly performed using alloplastic facial implants (1–3). A wide range of alloplastic implant materials have been used for these purposes of which titanium, porous polyethylene (MedPor), polyether-ether-ketone (PEEK), silicone and poly-methyl methacrylate (PMMA) are among the most used materials. Each material possesses physicochemical properties and biological profiles, with associated advantages and risks (1, 4, 5).

Facial implants are used to address bony defects, for example resulting from trauma, oncologic resections and congenital deficiencies (1–3). Among these, oncologic resections account for a relevant portion of cases requiring facial reconstruction. In 2020, an estimated 930,000 new cases of head and neck cancers were reported worldwide, including cancers of the lip and oral cavity, salivary glands, oropharynx, nasopharynx, hypopharynx, and larynx, according to GLOBOCAN 2020 estimates from the International Agency for Research on Cancer (IARC) (4). These estimates underscore the substantial burden of head and neck malignancies globally. Other patients who may need implant-based reconstruction are facial trauma patients (5). In 2017 alone, there were an estimated 7.5 million new cases of facial fractures globally, based on data from the Global Burden of Disease Study (6). In addition to reconstructive indications, implants are frequently used for aesthetic facial augmentations, such as chin and midfacial enhancements (2, 3). According to the 2023 ASPS procedural statistics report the number of cheek implants increased by 7% to 8,825 procedures in 2023, while chin implant procedures rose by 1% to 5,484 cases, reflecting the rising interest in facial augmentation (7).

To date, there is insufficient evidence to establish the superiority of one specific material for use in facial implantology. Outcome reports are often limited to case series with short follow-ups. Additionally, patient-specific implants are increasingly used as opposed to standard “off-the-shelf” implants. The added benefit of anatomical customization has not been systematically evaluated.

This systematic review of the literature comprehensively summarizes the experience with facial alloplastic implants over the last 54 years (1970–2024), aiming to provide an update on the risk profile of selected implant materials and to help guide evidence-based treatment decision making.

## Methods

**Figure 1:**
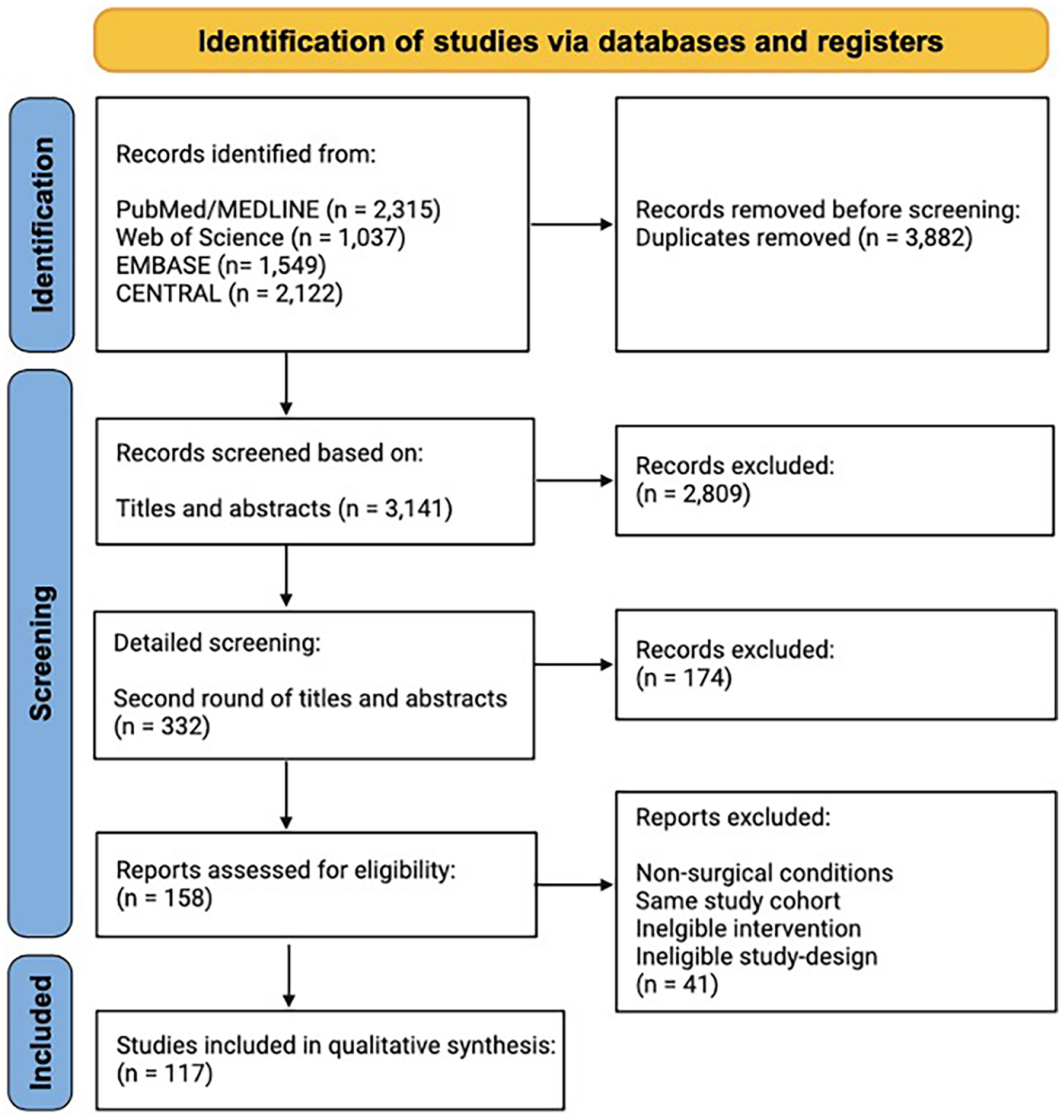
PRISMA 2020 flow diagram of the study identification process.

This study was conducted in accordance with the Preferred Reporting Items for Systematic Reviews and Meta-Analyses (PRISMA) 2020 (6). This study should be viewed as a descriptive review, as we did not perform a meta-analysis due to the heterogeneity observed in outcome parameters. This systematic review was registered with the International Prospective Register of Systematic Reviews (PROSPERO identifier: CRD42024501754). A detailed description of the search strategy and search string can be found in the Supplementary Digital Content; Figure 1 (including Prisma 2020 flowchart). In terms of inclusion and exclusion criteria, we only included studies that used an alloplastic material to permanently replace missing parts of the splanchnocranium (reconstruction) or for augmentation for aesthetic purposes. Other alloplastic materials/implant materials used for skeletal fixation (e.g., titanium plates or absorbable plates made from polyglycolic acid), injectable fillers (e.g., dermal injectable hydroxyapatite fillers such as Radiesse) and implants used for the reconstruction of the neurocranium were excluded. Quality assessment of the included studies was conducted using the Newcastle Ottawa Score (NOS) and the Level of Evidence (LOE) (Supplementary Tables 2, 3).

### Data extraction

The following data points were extracted: Digital object identifier (DOI), first author, journal, publication year, study population size, study design, implant material, material costs [USD], mechanical material properties, printing technology, facial area targeted, clinical indication for implantation (i.e., reconstruction, aesthetic facial contouring), length of follow-up, acute/longer-term complications, surgical revision rate, implant acceptance rate, functional/aesthetic outcome, patient satisfaction, time from implant design to implantation [days], implant measurements [mm], antibiotic/anti-infectious prophylaxis. FIs were defined as PSIs when their manufacturing technique involved CT-based design and CAD/CAM or 3D printing (7).

## Results

### General study parameters

**Figure 2:**
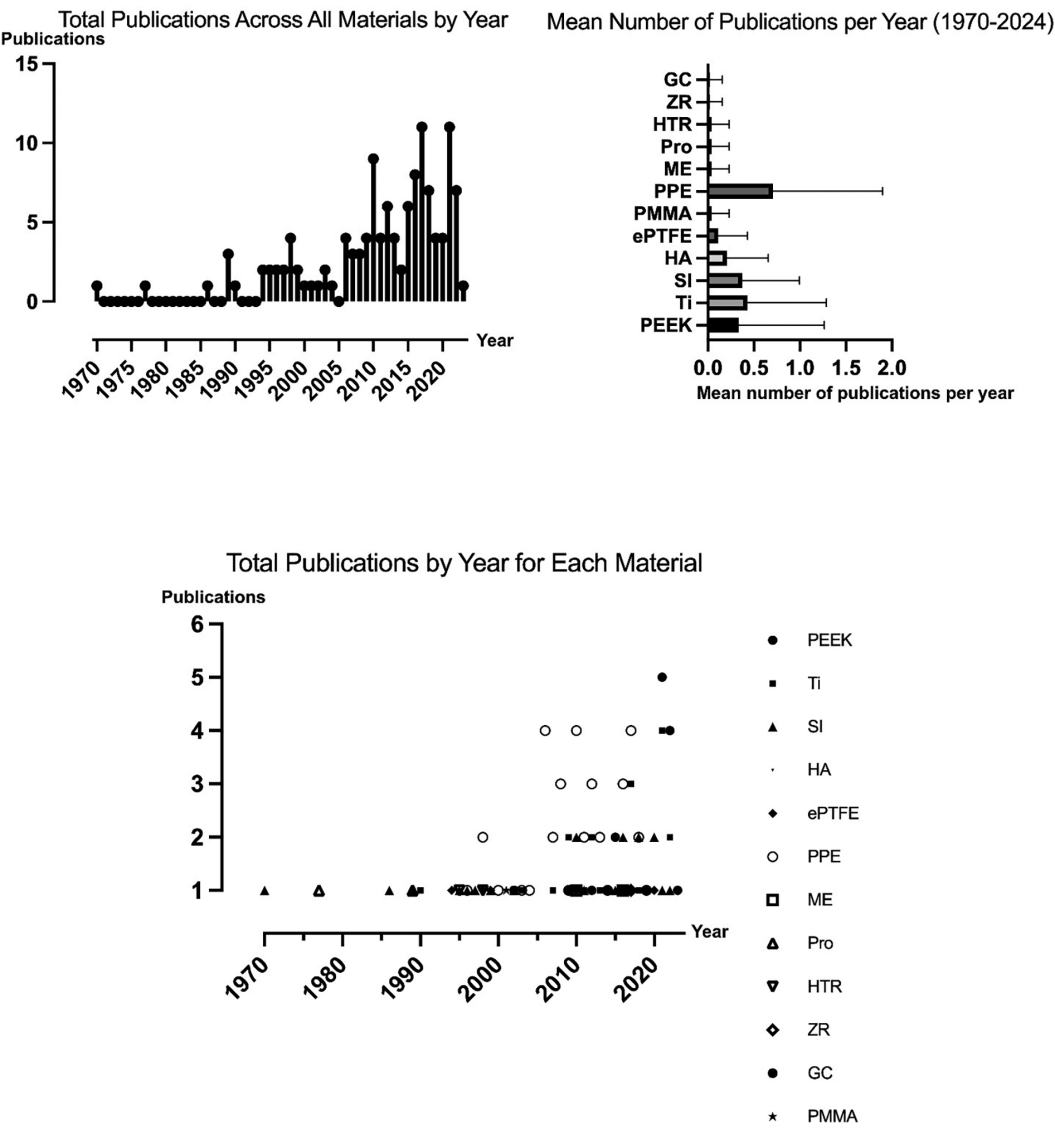
Trends in facial implantology publications: total publications and mean publications per material per year.

117 articles met the inclusion criteria totaling 4,273 patients (Table 1). Mean follow-up was 34 months (15 days to 25 years) (81, 101). In general, 66 articles (56%) investigated FIs for reconstructive indications, whereas 39 studies (33%) studied FIs to improve the patient's facial aesthetics. Overall, 12 studies (10%) investigated FI in both reconstructive and aesthetic procedures. Figure 2 illustrates the trend in the number of publications on facial implantology over time.

**Table 1:** General data of all included articles.

Study	Sample size	Patient age	Material	Fabrication technique	Length of follow up
Saporano et al. (8)	28	17 years (16–45 years)	PEEK	PSI	5 years
Nocini et al. (9)	1	25 years	PEEK	PSI	12 months
Shi et al. (10)	1	37 years	PEEK	PSI	12 months
Lim et al. (11)	10	32 years (9–78 years)	TI	PSI	37 ± 20 months
Ha et al. (12)	1	–	PEEK	PSI	–
Mayo et al. (13)	1	19 years (18–19 years)	TI	PSI	–
Kim et al. (14)	2	39 years (17–51 years)	SI	–	2 years
Hamsho et al. (15)	1	32 years	PEEK	PSI	6 months
Darwich et al. (16)	1	29 years	TI	PSI	18 months
Watanabe et al. (17)	207 (HA: 133, SI: 47)	28 years	HA, SI	OTS	9 months (6–59 months)
Ramieri (18)	1	40 years	PEEK	PSI	12 months
Antúnez-Conde et al. (19)	1	55 years	TI	PSI	–
Olate et al. (20)	1	25 years	PEEK	PSI	12 months
Sesqué et al. (21)	1	50 years	TI	-	12 months
Yashin et al. (22)	5	39 years (35–44 years)	TI	PSI	–
Khashaba, (23)	10	42 years (29–67 years)	PEEK	PSI	12 months
Narciso et al. (24)	1	50 years	PEEK	PSI	12 months
Yang et al. (25)	2	47 years (45–49 years)	PEEK	PSI	32 months
Bai et al. (26)	200	15–39 years	SI, ePTFE	OTS	6 months
Jang et al. (27)	60	39 years	HA/PLLA, PCL	–	6 months
Mrad et al. (28)	1	39 years	SI	–	3 months
Doh et al. (29)	1	53 years	PEEK	PSI	>6 days
Scofield-Kaplan et al. (30)	2	52years (42–61 years)	TI, PPE	OTS	6–9 months
Tsumiyama et al. (31)	72	36 years (7–74 years)	HA/PLLA	OTS	292 days (113–811 days)
Suh et al. (32)	1	68 years	PPE	–	5 years
Woo et al. (33)	1	20 years	TI	PSI	–
Findikcioglu et al. (34)	3	27 years (22–33 years)	PPE	OTS	22–34 months
Kanazawa et al. (35)	1	6 years	HA	–	10 years
Al-Jandan and Marei (36)	58	–	SI	–	12 months
Sciaraffia et al. (37)	15	34 years (14–57 years)	SI	OTS	12 mo-17 years
Kohyama et al. (38)	70	45 ± 22 years (5–84 years)	HA/PLLA	OTS	30 ± 13 months (3–52 months)
Franco et al. (39)	1	19 years	PPE	–	6 months
Zieliński et al. (40)	93	39 ± 17 years	TI, PPE, ZR	PSI, OTS	6 months
Callahan et al. (41)	5	11–75 years	TI, PPE	PSI	11 months
Sainsbury et al. (42)	3	11 years (7–16 years)	PEEK	PSI	–
Cho et al. (43)	1	22 years	PPE	OTS	12 years
Lee et al. (44)	2	53 years (40–66 years)	TI	–	2 months
Ghosh 2017 (45)	1	35 years	PMMA	–	–
Kanno, (46)	5	27 years	HA/PLLA	OTS	9 months (6–18 months)
Hosseini et al. (47)	1	14 years	PPE	–	4 –
Joo and Jang (48)	176	ePTFE: 30 years, ACC: 36 years (11–69 years)	ePTFE	OTS	12 months
Timoney et al. (49)	2	46 years (37–55 years)	PPE	–	2–16 –
Hussain, (50)	1	67 years	PEEK	PSI	“few days”
da Silva de Menezes et al. (51)	1	27 years	PPE	–	2 years
Park et al. (52)	2	24 years (23–24 years)	SI	–	7 months
Polo (53)	4	34 years (32–39 years)	SI, ME	–	10–17 years
Lavie et al. (54)	1	63 years	PEEK	PSI	15 months
Nahumi et al. (55)	1	13 years	PEEK	PSI	2 months
Gander et al. (56)	12	–	TI	PSI	–
Yim et al. (57)	3	42 ± 22 years (23–82 years)	SI	–	14 months
Rotaru et al. (58)	1	43 years	TI	PSI	12 months
Park et al. (59)	10	37 ± 14 years	HA/PLLA	OTS	2 months
Jalbert et and Haers (60)	5	50 years (30–69 years)	PEEK	PSI	3–12 months
Atherton et al. (61)	3	14 years (14–15 years)	PPE	–	24 months
Kozakiewicz et al. (62)	57	34 ± 14 years	TI, PPE	PSI	6 months
Kozakiewicz et al. (63)	1	–	PPE	PSI	6 months
Alonso et al. 2013 (64)	1	54 years	PPE	–	10 years
Hatamleh et al. 2013 (65)	4	35 years (18–49 years)	TI	PSI	5 years
Hayashi et al. 2013 (66)	17	40 years (10–80 years)	HA/PLLA	OTS	22 months ± 15 months (6–60 months)
Guo et al. (67)	102	18–65 years	TI	OTS	6–24 months
Kim et al. (68)	42	31 years (13–74 years)	PPE (PPCI), TI (PPTB)	–	3 months
Scolozzi et al. (69)	2	18–25 years	PEEK	PSI	2 years
Niechajev (70)	102	27 years (18–70 years)	PPE	OTS	7 years (6 months-15 years)
Lin and Chen (71)	95	18–42 years	PPE	OTS	35 months (3–51 months)
Kirby et al. (72)	317 (TI, PPE: 169)	34 years (14–85 years)	TI, PPE	–	39 weeks
Kim et al. (73)	55	29 years	PPE	OTS	9.4 months
Aynehchi et al. (74)	125	31 years (18–56 years)	SI	OTS	17 months
Atherton et al. (75)	10	40 years (25–56-years)	ME	OTS	6–38 months
Park et al. (76)	19	22 years (18–37 years)	PPE	–	6 months
Li et al. (77)	9 (PPE: 3, SI: 3, ePTFE: 3)	18–40 years	PPE, SI, ePTFE	OTS	6–12 months
Chen et al. (78)	32	22 years (16–31 years)	PPE	–	25.4 months
Tang et al. (79)	46	38.7 years	TI	PSI	6–12 months
Hopping et al. (80)	100	–	SI	OTS	12–48 months
Deshpande et al. (81)	44	25 years (14–58 years)	PPE	OTS	46 months (15 days–100 months
Kim et al. (82)	4	19 years (11–29 years)	PEEK	–	4 months
Stringer and Brown (83)	5	18–45 years	TI	–	10 months–15 years
Jirman et al. (84)	1	30 years	PPE	PSI	6 months
Guo et al. (85)	61	38 years (21–65 years)	TI	–	8–22 months
Emsen and Benlier (86)	1	6 months	PPE	OTS	2 years
Gui et al. (87)	150	24 years (20–27 years)	PPE	–	6 months–6 years
Coban and Kabalci (88)	3	–	PPE	OTS	–
Garibaldiet al. (89)	106	31 years (3–85 years)	PPE	–	3.5 months
Scholz et al. (90)	1	16 years	TI	PSI	4 weeks
Eski et al. (91)	22	–	PPE	OTS	13 months (6–24 months)
Ozturk et al. (92)	1	20 years	PPE	–	1 months
Romo and Kwak (93)	1	45 years	PPE	–	12 months
Gürlek et al. (94)	20	31 years (21–48 years)	PPE	OTS	18 months
Thornton and Mendelsohn (95)	37	39 years (17–65 years)	PPE	–	–
Menderes et al. (96)	71	–	PPE	OTS	12 months
Ellis and Tan (97)	58 (TI: 32)	32 years (16–58 years)	TI	–	–
Yaremchuck (98)	162	31 years (12–72 years)	PPE	OTS	27 months (0–132 months)
Duskováet al. (99)	44	25 years (16–54 years)	GC	–	16–48 months
Saleh et al. (100)	40	29 ± 9 years (16–50 years)	SI	OTS	23 months (9–60 months)
Lustica (140)	19	32 years (21–69 years)	PMMA	–	<25 years
Sevin et al. (102)	31	21 years (5–34 years)	PPE	–	8–9 So
Ramirez et al. (103)	13	47–54 years	PPE	OTS	–
Yaremchuck (104)	11	30 years (21–52 years)	PPE	OTS	2 years (3 months–4 years)
Fedok et al. (105)	5	46 years (19–81 years)	ePTFE	OTS	4–20 months
Metzinger et al. (106)	60	47 years (17–76 years)	SI	OTS	12 months
Mendelsohn and Dunlop (107)	30	–	ePTFE	–	<18 years
Karras and Wolford (108)	18	26 years (14–44 years)	HTR (PMMA + HA)	OTS	21.5 months
Yaremchuk and Israeli (109)	9	35 years (23–48 years)	PPE	OTS	33 months
Frodel and Lee (110)	34	20–74 years	PPE	OTS	6–40 months
Hirano et al. (111)	2	44 years (42–46 years)	HA	OTS	8 months–4 years
–Abrahams and Caceres (112)	4	–	SI	–	–
Semergidis et al. (113)	18	43–58 years	PPE	–	6–36 months
Vuyk (114)	40	28 years (19–50 years)	SI	OTS	1–45 months
Eppley et al. (115)	61	9–37 years	HTR	OTS	2–5 years
Matarasso et al. (116)	6	22–62 years	SI	–	4–30 years
Owsley and Taylor (117)	106	22–44 years	ePTFE	OTS	5 years
Ono et al. (118)	11	22 years (19–25 years)	HA	PSI	10–31 months
Blake et al. (119)	20	28 years (2–60 years)	TI	PSI	–
Moenning and Wolford (120)	62 (PRO: 50, HA: 12)	24 years (12–54 years)	HA, PRO	OTS	45 mo (PI implants), 33 mo (PII implants), 19 mo (PBHA implants)
Epker and Stella (121)	15	22 years (18–37 years)	SI	–	8 years
Pitanguy et al. (122)	612 (S: 601, AC: 11)	–	SI, AC	OTS	16 years
Dann and Epker (123)	31	19.2 yrs	PRO	–	–
Laub et al. (124)	25	23 years (20–28 years)	SI	OTS	6 months
	*N* = 4,273	Mean: 33 years	**487 (TI)** **65 (PEEK)** **1,039 (PPE)** **320 (ePTFE)** **1,303 (SI)** **10 (ME)** **393 (HA/PLLA)** **60 (PCL)** **20 (PMMA)** **44 (GC)** **31 (PRO)** **93 (ZR) + 79 (HTR)**	**332 (PSI) + 2.651 (OTS)**	**Mean: 34 months**
			**2.641** ^a^	**2.983** ^a^	

Bold values indicate total numbers.

^a^ Some articles reported on several regions or did not report on the implant fabrication techniques. Therefore, the total number of patients may differ; PSI, patient-specific implant; OTS, off-the-shelf implant; TI, titanium; PEEK, polyetheretherketone; PPE, polyethylene; ePTFE, expanded polytetrafluorethylene; SI, silicone; ME, mersilene; HA/PLLA, hydroxylapatite/poly-l-lactide; PCL, polycaprolactone; PMMA, polymethylmethacrylate; GC, glass ceramics; PRO, Proplast; ZR, zirconium oxide; HTR, hard tissue replacement.

Of the 117 studies included, 34 studies (29%) focused on FIs made of porous polyethylene, 18 (15%) on FIs made of titanium, 18 (15%) on FIs made of polyetheretherketone (PEEK), and 15 (13%) on articles investigating silicone facial implants. Eight studies (6.8%) investigated FIs made of hydroxylapatite (HA), of which six (5.1%) addressed hydroxylapatite/poly-l-lactide (HA/PLLA) FIs. Four studies (3.4%) investigated polytetrafluoroethylene (ePTFE) implants, respectively, while two studies (1.7%) studied FIs made of polymethylmethacrylate (PMMA). One article (0.85%) each focused on Proplast implants, hard tissue replacement (HTR) implants, mersilene and glass ceramic. Additionally, 14 articles (12%) included multiple implant materials, of which titanium and polyethylene were the most common combination (*n* = 6; 5.1%).

**Figure 3:**
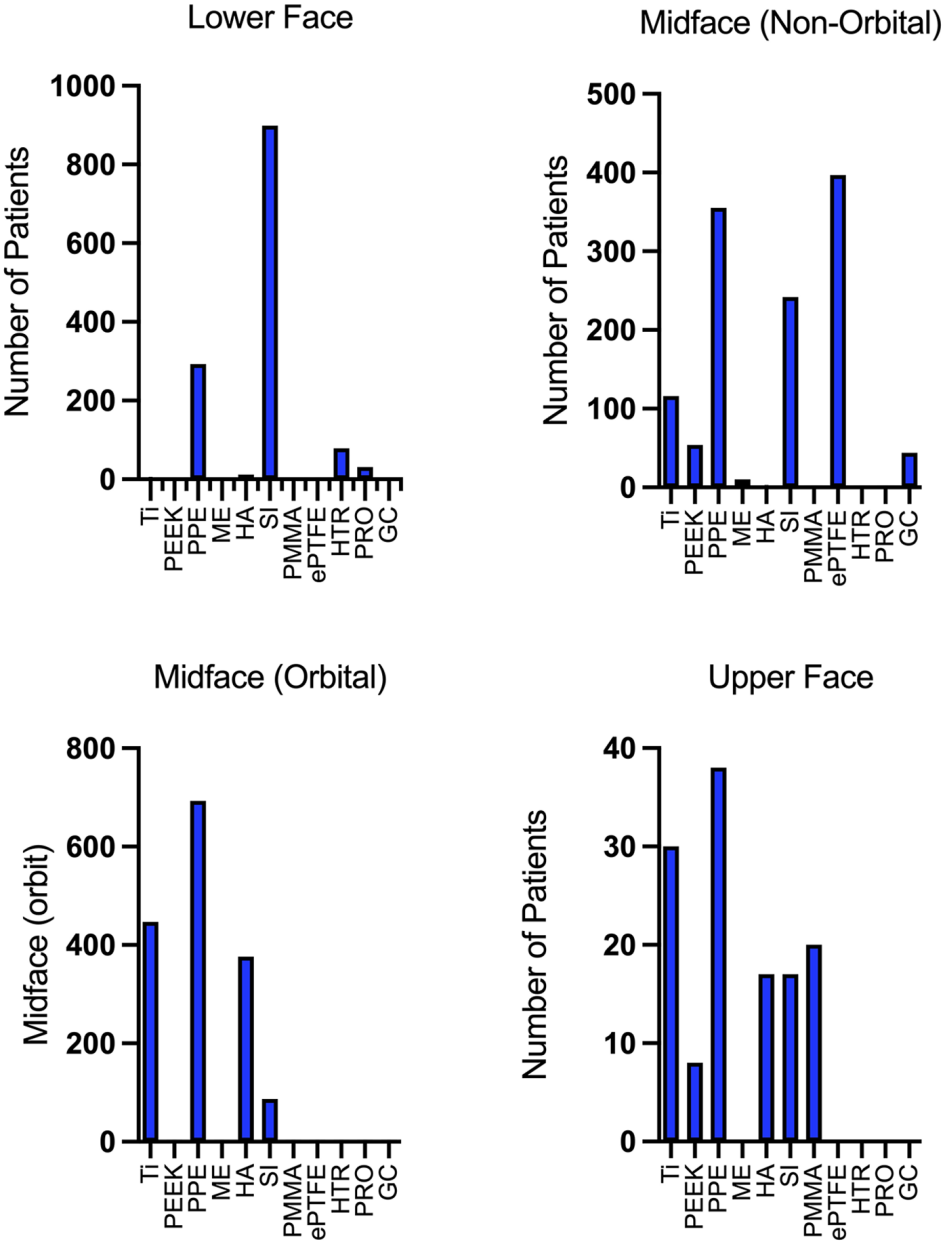
Patient distribution by facial implant material and anatomical region.

Patient ages ranged from 6 months to 85 years. Most studies focused on the orbital (*n* = 34; 29%), chin (*n* = 26, 22%), and malar region (*n* = 22; 19%) (Table 1; Figure 3).

### Implant manufacturing

In 34 articles (29%), authors described manufacturing techniques of FIs based on preoperative scans [i.e., patient-specific implants (PSIs)] (7, 9–25). In contrast, 43 studies (36%) investigated the utilization of prefabricated implants (i.e., off-the-shelf implants) (29–115). Of all patients receiving off-the-shelf implants, 6.5% (175/2,684) showed complications, while the total complication rate of PSI-treated patients was 1.6% (7/435). Detailed complication rates ordered by fabrication technique are shown in Table 2.

**Table 2:** Complication rates of patient-specific implants (PSIs) and off-the-shelf (OTS) implants per patient in the upper face (FH, OF, FN), the orbital region (ORB), the midface (MAL, NOSE, PN, MAX, TEMP), and the lower face (CHIN, MAN).

	PSI	OTS	PSI	OTS	PSI	OTS	PSI	OTS
Upper face								
**Total number of patients**	**38**	**52**	**1**	**35**	**29**	**0**	**0**	**0**
Total complication rate [%]	2 (5.3)	5 (9.6)	0	5 (14)	0	0	0	0
Infection/inflammation [%]	0	1 (1.9)	0	1 (2.9)	0	0	0	0
Hematoma [%]	0	0	0	0	0	0	0	0
Persistent pain [%]	0	0	0	0	0	0	0	0
Nerve injury [%]	0	0	0	0	0	0	0	0
Wound dehiscence [%]	0	0	0	0	0	0	0	0
Implant exposure [%]	0	4 (7.7)	0	4 (7.7)	0	0	0	0
Displacement/extrusion [%]	0	0	0	0	0	0	0	0
Bone resorption [%]	0	0	0	0	0	0	0	0
Bone erosion [%]	0	0	0	0	0	0	0	0
Midface – orbital								
**Total number of patients**	**225**	**632**	**156**	**244**	**213**	**2**	**0**	**87**
Total complication rate [%]	0	33 (5.2)	0	6 (2.5)	0	0	0	9 (10)
Infection/inflammation [%]	0	5 (0.79)	0	4 (1.6)	0	0	0	1 (1.1)
Hematoma [%]	0	3 (0.47)	0	0	0	0	0	2 (2.3)
Persistent pain [%]	0	0	0	0	0	0	0	0
Nerve injury [%]	0	25 (4.0)	0	2 (0.82)	0	0	0	6 (6.9)
Wound dehiscence [%]	0	0	0	0	0	0	0	0
Implant exposure [%]	0	0	0	0	0	0	0	0
Displacement/extrusion [%]	0	0	0	0	0	0	0	0
Bone resorption [%]	0	0	0	0	0	0	0	0
Bone erosion [%]	0	0	0	0	0	0	0	0
Midface - non-orbital								
**Total number of patients**	**169**	**907**	**0**	**248**	**116**	**0**	**0**	**240**
Total complication rate [%]	5 (3.0)	31 (3.4)	0	11 (4.4)	1 (0.86)	0	0	20 (8.3)
Infection/inflammation [%]	0	13 (1.4)	0	4 (1.6)	0	0	0	8 (3.3)
Hematoma [%]	1 (0.59)	3 (0.33)	0	0	0	0	0	2 (0.83)
Persistent pain [%]	0	1 (0.11)	0	0	0	0	0	1 (0.42)
Nerve injury [%]	0	5 (0.55)	0	0	0	0	0	4 (1.7)
Wound dehiscence [%]	0	0	0	0	0	0	0	0
Implant exposure [%]	0	2 (0.22)	0	2 (0.22)	0	0	0	0
Displacement/extrusion [%]	4 (2.4)	7 (0.77)	0	5 (2.0)	1 (0.86)	0	0	5 (2.1)
Bone resorption [%]	0	0	0	0	0	0	0	0
Bone erosion [%]	0	0	0	0	0	0	0	0
Lower face								
**Total number of patients**	**3**	**1,093**	**0**	**125**	**1**	**0**	**0**	**824**
Total complication rate [%]	0	106 (9.7)	0	2 (1.6)	0	0	0	98 (12)
Infection/inflammation [%]	0	3 (0.27)	0	1 (0.80)	0	0	0	0
Hematoma [%]	0	2 (0.18)	0	0	0	0	0	2 (2.4)
Persistent pain [%]	0	2 (0.18)	0	0	0	0	0	0
Nerve injury [%]	0	49 (4.5)	0	0	0	0	0	49 (5.9)
Wound dehiscence [%]	0	2 (0.18)	0	0	0	0	0	0
Implant exposure [%]	0	0	0	0	0	0	0	0
Displacement/extrusion [%]	0	5 (0.46)	0	1 (0.80)	0	0	0	4 (4.9)
Bone resorption [%]	0	29 (2.7)	0	0	0	0	0	29 (3.5)
Bone erosion [%]	0	14 (1.3)	0	0	0	0	0	14 (1.7)

PSI, patient-specific implant; OTS, off-the-shelf implant; FH, forehead; MF, midface; OF, orbitofrontal; FN, frontonasal; ORB, orbital; NOSE, nose; PN, paranasal; MAX, maxilla; MAN, mandible, MAL, malar; CHIN, chin.

Bold values indicate total numbers.

### General intra- and postoperative complications

Out of 117 articles, 50 (43%) did not report any intraoperative or postoperative complications during the follow-up period. The mean follow-up period of studies reporting no complications was 25 months. The majority of articles without any complications included polyethylene implants (*n* = 18; 15%), followed by PEEK implants (*n* = 12; 10%) and titanium implants (*n* = 11; 9.3%) (8–11, 15, 16, 18–21, 24, 25, 29, 30, 39, 41, 42, 47, 51, 58, 61, 65, 67, 69, 83, 84, 86–88, 90–95, 109, 113, 119). When excluding case reports and case series with less than five patients (ECR), the highest complication rate was 58% (54). Revision rates ranged from 0% to 26%, while 35 articles (30%) reported revision surgeries (ECR) (3, 11, 19, 31, 32, 36, 39, 43, 49, 50, 58, 66, 70, 71, 75, 78, 80, 82, 87, 89, 95, 96, 98, 99, 101, 102, 104–107, 112, 114–116, 121, 122, 125, 126). In total, 190 patients showed complications, yielding a complication rate per patient of 4.4% [(190/4,273) for details see Tables 3A–D].

**Table 3A:** Complication rates per patient for FIs of the upper face (FH, OF, FN).

Implant material	TI	PEEK	PPE	ME	HA(/PLLA)	SI	PMMA	ePTFE	HTR	PRO	GC	PCL
**Total number of patients**	**30**	**8**	**38**	**0**	**17**	**17**	**20**	**0**	**0**	**0**	**0**	**0**
Total complication rate [%]	0	0	5 (13)	0	0	2 (12)	2 (10)	0	0	0	0	0
Infection/inflammation [%]	0	0	1 (2.6)	0	0	2 (12)	1 (5)	0	0	0	0	0
Hematoma [%]	0	0	0	0	0	0	1 (5)	0	0	0	0	0
Persistent pain [%]	0	0	0	0	0	0	0	0	0	0	0	0
Nerve injury [%]	0	0	0	0	0	0	0	0	0	0	0	0
Wound dehiscence [%]	0	0	0	0	0	0	0	0	0	0	0	0
Implant exposure [%]	0	0	4 (11)	0	0	0	0	0	0	0	0	0
Displacement/extrusion [%]	0	0	0	0	0	0	0	0	0	0	0	0
Bone resorption [%]	0	0	0	0	0	0	0	0	0	0	0	0
Bone erosion [%]	0	0	0	0	0	0	0	0	0	0	0	0

TI, titanium; PEEK, polyetheretherketone; PPE, polyethylene; ePTFE, expanded polytetrafluorethylene; SI, silicone; ME, mersilene; HA/PLLA, hydroxylapatite/poly-l-lactide; PCL, polycaprolactone; PMMA, polymethylmethacrylate; GC, glass ceramics; PRO, Proplast; ZR, zirconium oxide; HTR, hard tissue replacement; FH, forehead; MF, midface; OF, orbitofrontal; FN, frontonasal; ORB, orbital; NOSE, nose; PN, paranasal; MAX, maxilla; MAN, mandible, MAL, malar; CHIN, chin.

Bold values indicate total numbers.

**Table 3B:** Complication rates per patient for FIs of the orbital region (ORB).

Implant material	TI	PEEK	PPE	ME	HA (/PLLA)	SI	PMMA	ePTFE	HTR	PRO	GC	PCL
**Total number of patients**	**447**	**0**	**693**	**0**	**376**	**87**	**0**	**0**	**0**	**0**	**0**	**0**
Total complication rate [%]	46 (10)	0	52 (7.5)	0	26 (6.9)	9 (10)	0	0	0	0	0	0
Infection/inflammation [%]	10 (2.2)	0	14 (2.0)	0	0	1 (1.1)	0	0	0	0	0	0
Hematoma [%]	0	0	0	0	3 (0.80)	2 (2.3)	0	0	0	0	0	0
Persistent pain [%]	8 (1.8)	0	8 (1.2)	0	0	0	0	0	0	0	0	0
Nerve injury [%]	25 (5.6)	0	27 (3.9) (2)	0	23 (6.1)	6 (6.9)	0	0	0	0	0	0
Wound dehiscence [%]	0	0	0	0	0	0	0	0	0	0	0	0
Implant exposure [%]	0	0	0	0	0	0	0	0	0	0	0	0
Displacement/extrusion [%]	3 (0.67)	0	3 (0.43)	0	0	0	0	0	0	0	0	0
Bone resorption [%]	0	0	0	0	0	0	0	0	0	0	0	0
Bone erosion [%]	0	0	0	0	0	0	0	0	0	0	0	0

TI, titanium; PEEK, polyetheretherketone; PPE, polyethylene; ePTFE, expanded polytetrafluorethylene; SI, silicone; ME, mersilene; HA/PLLA, hydroxylapatite/poly-l-lactide; PCL, polycaprolactone; PMMA, polymethylmethacrylate; GC, glass ceramics; PRO, Proplast; ZR, zirconium oxide; HTR, hard tissue replacement; FH, forehead; MF, midface; OF, orbitofrontal; FN, frontonasal; ORB, orbital; NOSE, nose; PN, paranasal; MAX, maxilla; MAN, mandible, MAL, malar; CHIN, chin.

Bold values indicate total numbers.

**Table 3C:** Complication rates per patient for FIs of the midface except for the orbital area (MAL, NOSE, PN, MAX, TEMP).

Implant material	TI	PEEK	PPE	ME	HA(/PLLA)	SI	PMMA	ePTFE	HTR	PRO	GC	PCL
**Total number of patients**	**116**	**54**	**355**	**10**	**3**	**242**	**0**	**397**	**0**	**0**	**44**	**0**
Total complication rate [%]	1 (0.86)	1 (1.9)	15 (4.2)	1 (10)	0	20 (8.3)	0	5 (1.3)	0	0	10 (23)	0
Infection/inflammation [%]	0	0	5 (1.4)	1 (10)	0	8 (3.3)	0	3 (0.76)	0	0	0	0
Hematoma [%]	0	0	0	0	0	2 (0.83)	0	1 (0.25)	0	0	1 (2.3)	0
Persistent pain [%]	0	0	0	0	0	1 (0.41)	0	0	0	0	0	0
Nerve injury [%]	0	0	0	0	0	4 (1.7)	0	1 (0.25)	0	0	0	0
Wound dehiscence [%]	0	1 (1.9)	0	0	0	0	0	0	0	0	0	0
Implant exposure [%]	0	0	5 (1.4)	0	0	0	0	0	0	0	0	0
Displacement/extrusion [%]	1 (0.86)	0	5 (1.4)	0	0	5 (2.1)	0	0	0	0	9 (20)	0
Bone resorption [%]	0	0	0	0	0	0	0	0	0	0	0	0
Bone erosion [%]	0	0	0	0	0	0	0	0	0	0	0	0

TI, titanium; PEEK, polyetheretherketone; PPE, polyethylene; ePTFE, expanded polytetrafluorethylene; SI, silicone; ME, mersilene; HA/PLLA, hydroxylapatite/poly-l-lactide; PCL, polycaprolactone; PMMA, polymethylmethacrylate; GC, glass ceramics; PRO, Proplast; ZR, zirconium oxide; HTR, hard tissue replacement; FH, forehead; MF, midface; OF, orbitofrontal; FN, frontonasal; ORB, orbital; NOSE, nose; PN, paranasal; MAX, maxilla; MAN, mandible, MAL, malar; CHIN, chin.

Bold values indicate total numbers.

**Table 3D:** Complication rates per patient for FIs of the lower face (CHIN, MAN).

Implant material	TI	PEEK	PPE	ME	HA(/PLLA)	SI	PMMA	ePTFE	HTR	PRO	GC	PCL
**Total number of patients**	**6**	**2**	**293**	**4**	**12**	**899**	**0**	**3**	**79**	**31**	**0**	**0**
Total complication rate [%]	0	0	51 (17)	0	0	70 (7.8)	0	0	6 (7.6)	12 (39)	0	0
Infection/inflammation [%]	0	0	1 (0.34)	0	0	3 (0.33)	0	0	2 (2.5)	2 (6.5)	0	0
Hematoma [%]	0	0	49 (17)	0	0	2 (0.22)	0	0	0	0	0	0
Persistent pain [%]	0	0	0	0	0	1 (0.11)	0	0	2 (2.5)	0	0	0
Nerve injury [%]	0	0	0	0	0	0	0	0	0	4 (13)	0	0
Wound dehiscence [%]	0	0	0	0	0	0	0	0	2 (2.5)	6 (19)	0	0
Implant exposure [%]	0	0	0	0	0	0	0	0	0	0	0	0
Displacement/extrusion [%]	0	0	1 (0.34)	0	0	12 (1.3)	0	0	0	0	0	0
Bone resorption [%]	0	0	0	0	0	29 (3.2)	0	0	0	0	0	0
Bone erosion [%]	0	0	0	0	0	23 (2.6)	0	0	0	0	0	0

TI, titanium; PEEK, polyetheretherketone; PPE, polyethylene; ePTFE, expanded polytetrafluorethylene; SI, silicone; ME, mersilene; HA/PLLA, hydroxylapatite/poly-l-lactide; PCL, polycaprolactone; PMMA, polymethylmethacrylate; GC, glass ceramics; PRO, Proplast; ZR, zirconium oxide; HTR, hard tissue replacement; FH, forehead; MF, midface; OF, orbitofrontal; FN, frontonasal; ORB, orbital; NOSE, nose; PN, paranasal; MAX, maxilla; MAN, mandible, MAL, malar; CHIN, chin.

Bold values indicate total numbers.

### Complication rates ordered by implant material

This analysis excludes case reports and series with fewer than five patients to provide more robust assessment of complication rates across various implant materials. The rate of complications was highest (48%) in a study investigating polyethylene FIs for mental reconstruction (71). The highest infection rate was found for temporal implants made of mersilene (10%) (75). Hematomas rates peaked at 5.3% in a study that examining PMMA FIs for fronto-orbital reconstruction (101). Rates of persistent pain following FI were reported to be as high as 10% in one study using titan and polyethylene implants for the reconstruction of orbital floor fractures (72). A study on aesthetic chin surgery using polyethylene FI revealed transient nerve injuries in 48% of cases (71). The rate of wound dehiscence was highest (20%) in an article investigating Proplast implants for chin augmentation (123). Implant exposure rates reached up to 12% in a study using polyethylene implants for reconstruction of facial deformities, respectively (110). The rate of implant displacement was as high as 8.3% in a study on silicone implants for the malar region (106). Further, the rate of implant extrusion was as high as 20% in a study reporting on the use of glass ceramics (99). One study on aesthetic chin augmentation using silicone FI revealed bone resorption in 53% of cases (100). Another study on aesthetic genioplasty reported bone erosion in up to 93% (37).

Notably, 50 of 117 studies (43%) that reported on postoperative complications found no adverse events. The rate of complication-free postoperative recovery was highest for polyethylene FIs. Overall, 18 of 33 polyethylene studies (55%), that reported postoperative complication rates, had complication-free recovery. However, it is worth noting that for various implant materials (e.g., PCL) only one study looked into complications and did not report any complications (25–27, 117). Detailed complication rates ordered by implant material are listed in Table 4.

**Table 4:** Postoperative complications in various implant materials (ECR). [Percentage (number of affected patients/total number of patients with data on the respective complication)].

Implant material	TI	PEEK	PPE	ME	HA/PLLA	SI	PMMA	ePTFE	ZR	GC	PRO	HTR	PCL
Total complication rate [%]	0.00 (0/20)–17 (2/12)	0.00 (0/28)–50 (5/10)	0.00 (0/150)–48 (46/95)	10 (1/10)	0.00 (0/11)–19 (14/72)	0.00 (0/125)–24 (14/58)	0.00 (0/18)–3.3 (3/19)	0.00 (0/106)–20 (6/30)	–	23 (10/44)	39 (12/31)	0.00 (0/18)–10 (6/61)	0.00 (0/30)
Infection/inflammation [%]	0.00 (0/20)–5.9 (10/169)	0.00 (0/28)	0.00 (0/150)–7.7 (1/13)	10 (1/10)	0.00 (0/11)	0.00 (0/125)–8.0 (8/100)	0.00 (0/18)–5.3 (1/19)	0.00 (0/106)–10 (3/30)	–	0.00 (0/44)	6.5 (2/31)	0.00 (0/18)–3.3 (2/61)	0.00 (0/30)
Hematoma [%]	0.00 (0/20)	0.00 (0/28)	0.00 (0/150)	0.00 (0/10)	0.00 (0/11)	0.00 (0/125)–5.0 (2/40)	0.00 (0/18)–5.3 (1/19)	0.00 (0/106)	–	2.3 (1/44)	0.00 (0/31)	0.00 (0/18)	0.00 (0/30)
Persistent pain [%]	0.00 (0/20)–10 (16/169)	0.00 (0/28)	0.00 (0/150)–10 (16/169)	0.00 (0/10)	0.00 (0/11)	0.00 (0/125)–1.0% (1/100)	0.00 (0/18)	0.00 (0/106)	–	0.00 (0/44)	0.00 (0/31)	0.00 (0/18)	0.00 (0/30)
Nerve injury [%]	0.00 (0/20)–15 (25/169)	0.00 (0/28)	0.00 (0/150)–48 (46/95)	0.00 (0/10)	0.00 (0/11)–19 (14/72)	0.00 (0/125)–5.0% (3/60)	0.00 (0/18)	0.00 (0/106)	–	0.00 (0/44)	13 (4/31)	0.00 (0/18)	0.00 (0/30)
Wound dehiscence [%]	0.00 (0/20)	0.00 (0/28)	0.00 (0/150)–	0.00 (0/10)	0.00 (0/11)	0.00 (0/125)	0.00 (0/18)	0.00 (0/106)	–	0.00 (/44)	20 (6/31)	0.00 (0/18)–3.3 (2/61)	0.00 (0/30)
Implant exposure [%]	0.00 (0/20)	0.00 (0/28)	0.00 (0/150)–12 (4/34)	0.00 (0/10)	0.00 (0/11)	0.00 (0/125)	0.00 (0/18)	0.00 (0/106)	–	0.00 (0/44)	0.00 (0/31)	0.00 (0/18)	0.00 (0/30)
Displacement/extrusion [%]	0.00 (0/20)–3.5 (1/28)	0.00 (0/28)	0.00 (0/150)–7.7 (1/13)	0.00 (0/10)	0.00 (0/11)	0.00 (0/125)–14 (8/58)	0.00 (0/18)	0.00 (0/106)	–	20 (9/44)	0.00 (0/31)	0.00 (0/18)	0.00 (0/30)
Bone resorption [%]	0.00 (0/20)	0.00 (0/28)	0.00 (0/150)	0.00 (0/10)	0.00 (0/11)	0.00 (0/125)–53 (21/40)	0.00 (0/18)	0.00 (0/106)	–	0.00 (0/44)	0.00 (0/31)	0.00 (0/18)	0.00 (0/30)
Bone erosion [%]	0.00 (0/20)	0.00 (0/28)	0.00 (0/150)	0.00 (0/10)	0.00 (0/11)	0.00 (0/125)–93 (14/15)	0.00 (0/18)	0.00 (0/106)	–	0.00 (0/44)	0.00 (0/31)	0.00 (0/18)	0.00 (0/30)

[Percentage (number of affected patients/total number of patients with data on the respective complication)].

TI, titanium; PEEK, polyetheretherketone; PPE, polyethylene; ePTFE, expanded polytetrafluorethylene; SI, silicone; ME, mersilene; HA/PLLA, hydroxylapatite/poly-l-lactide; PCL, polycaprolactone; PMMA, polymethylmethacrylate; GC, glass ceramics; PRO, Proplast; ZR, zirconium oxide; HTR, hard tissue replacement.

### Infections and fistulas

Overall, 16 articles (14%) reported implant infections or inflammation as postoperative complications in 1.4%–10% of study cases (ECR) (36, 70, 72, 75, 78, 80, 81, 85, 98, 101, 103, 107, 110, 121, 123, 124, 127). In these studies, polyethylene (*n* = 7; 6.0%) and silicone (*n* = 4, 3.4%) were the most common implant materials, with infection rates between 1.4% and 8.0% (ECR) (36, 70, 78, 80, 81, 98, 103, 110, 121, 124). Postoperative cutaneous fistulas were described in two case reports using PPE with one patient each (1.7%) (43, 49, 64). In total, 44 patients exhibited infections, yielding a mean infection rate per patient of 1.0% (44/4,273).

### Hematoma

Seven studies (6.0%) reported postoperative hematoma, of which two articles (1.7%) addressed FIs made of silicone. One study (0.85%) each investigated FIs made of PEEK, HA/PLLA, HA, PTFE, glass ceramic, and PMMA. Rates of postoperative hematomas ranged between 1.0% and 5.0% (ECR) (17, 32, 38, 40, 50, 80, 89, 99, 101, 105, 114). 61 patients presented with postoperative hematoma, resulting in a cumulative hematoma rate per patient of 1.4% (61/4,273).

### Persistent postoperative pain

Persistent pain following implantation was reported in four studies (3.4%) (72, 80, 112, 115). The complication rates related to persistent pain ranged from 1.0% to 10%. Across all reviewed studies, 0.47% of patients (20/4,273) experienced persistent postoperative pain.

### Nerve injury

Eleven studies (9.4%) reported postoperative neuropraxia with paresthesia/hypesthesia, of which four articles (3.4%) used FIs made of PPE. Silicone was utilized in three studies (2.6%) and HA/PLLA in two studies (1.7%). Titanium, ePTFE, HA, HA/PLLA, and Proplast were each addressed in one study (0.85%) (17, 23, 31, 34, 38, 68, 71–73, 105, 106, 123). In total, 90 patients showed any nerve injury, yielding an overall rate of nerve injury per patient of 2.1% (90/4,273). Most nerve injuries affected branches of the trigeminal nerve (V3: 4 studies, V2: 6 studies). One study reported a case of temporal nerve paresis following temporal FI.

### Wound dehiscence and implant exposure

In three studies (2.6%), the authors reported postoperative wound dehiscence, while four articles (3.4%) revealed postoperative implant exposure, of which all occurred following the use of polyethylenic FIs. Implant exposure and wound dehiscence rates ranged between 3.1%–12% and 3.3%–19% (ECR), respectively (78, 81, 82, 102, 110, 115, 123). In sum, 0.21% (9 of 4,273 patients) patients with wound dehiscence were identified.

### Implant displacement

Nine studies (7.7%) reported on postoperative implant displacement or implant extrusion, ranging between 0.66% and 20% (ECR). Of these, four studies (3.4%) involved FIs made of polyethylene and three involved FIs made of silicone (2.6%) (36, 70, 72, 81, 103, 106, 122). Collectively, 25 patients showed postoperative implant displacement, yielding a total displacement rate per patient of 0.59% (25/4,273).

### Bone resorption/erosion

Six articles (5.1%) reported bone resorption (*n* = 2, 1.7%) or bone erosion (*n* = 4, 3.4%). All articles reported on silicone implants (37, 53, 100, 112, 114, 116). Bone resorption occurred in 29 out of 4,273 patients (0.68%) and bone erosion in 23 out of 4,273 patients (0.54%).

### Aesthetic and functional outcomes

Supplementary Table 4 presents detailed information on aesthetic outcomes. In brief, description of the aesthetic outcome varied widely but was most commonly described as “good” (*n* = 9; 7.7%), “excellent” (*n* = 7; 6.0%), or “improved” (*n* = 6; 5.1%). Reporting was heterogenous, with one study utilized an aesthetic outcome score ranging from 1 to 4 (1: unsatisfactory aesthetic outcome; 4: excellent aesthetic outcome) to assess the postoperative aesthetic outcomes (48). Two other studies used an analog visual scale (VAS), ranging from 0 to 10 (1: unsatisfactory outcome; 10: satisfactory outcome) to evaluate functional and aesthetic outcomes (11, 106). A different study compared implant projection to the mirrored contralateral side using Adobe Photoshop (Adobe Systems Incorporated, United States) to determine the side-to-side differences (60). 19 studies (16%) reported on poor aesthetic outcomes. Most of these studies investigated polyethylene FIs (*n* = 11; 9.4%). Again, there were no standardized outcome measurements (32, 39, 55, 70–72, 76, 80, 96, 98, 104, 106, 112, 114).

The authors revealed improved functional outcomes in 27 articles (23%), while eleven studies (9.4%) found poor functional outcomes. While improved functional outcomes encompassed a wide array of different functional parameters (e.g., improved airway function), all studies with unsatisfying functional results reported on eye bulb dysfunctions such as diplopia, persisting enophthalmos, or binocular vision loss (27, 40, 60, 62, 68, 72, 85, 91, 118, 126).

### Patient-reported outcomes and patient satisfaction

29 (25%) articles assessed patient satisfaction. Results were reported as “satisfied” (*n* = 13; 11%), “pleasing” (*n* = 5; 4.3%), or “excellent” (*n* = 1; 0.85%). Ten studies (8.5%) noted poor patient-reported outcomes, with dissatisfaction rates ranging from 0.16% to 16%. Most articles reporting satisfactory patient-reported outcomes used FIs made of polyethylene (*n* = 6; 5.1%) and silicone (*n* = 6; 5.1%) (9, 10, 24, 29, 39, 47, 74, 87, 91, 95, 100, 113). Further details on patient satisfaction are provided in Table 5.

**Table 5:** Patient satisfaction in various implant materials.

Implant material				
TI	PEEK	PPE	ME	SI
*“*significant improvement in the patient's psychological wellness and an increase in the quality of life” (16)	*“*satisfied” (9)	“pleased with the patients overall apperance” (39)	“pleasing cosmetic outcomes to surgeon and patient” (75)	“satisfied” (14)
*“*satisfactory aesthetic outcomes for surgeons and patients” (83)	*“*satisfied” (10)	“satisfied” (47)		dissatisfaction: 5.1% (3/58) (36)
	*“*satisfactory outcomes” (24)	7.8% (7/90) (dissatisfaction) (70)		“satisfied” (after swelling was gone) (52)
	*“*satisfied” (29)	good results: 94.7% (90/95), chin “too strong”: 4.2% (4/95), poor transition 1.1% (1/95) (71)		“satisfied” (57)
		patient-satisfaction rate: 84% (78)		“extremely satisfied” (74)
		66% (28/44): “extremely pleasing”, 29.5% (13/44): “pleasing”, 4.5% (2/44): “satisfactory”,2.3% (1/44): “not satisfactory” (81)		satisfaction rate was 96% [65% (65/100) very satisfied and 31% (31/100) satisfied], unsatisfied: 4.0% (4/100) (80)
		“satisfied” (87)		“happy” (100)
		“satisfied” (91)		85% (51/60): excellent, 8.3% (5/60): good, 1.7% (1/60): fair, 5.0% (3/60): poor (106)
		“happiness” (95)		“implant felt ‘natural’ and ‘a part of them’ indicating satisfactory stability”, “pleased” (114)
		1.9% (3/162) dissatisfaction (98)		0.16% (1/612) (dissatisfaction) (122)
		9.1% (1/11) asymmetry and irregularity (104)		
		“Satisfied”, “Excellent”, “Extremely Pleasing” (113)		

TI, titanium; PEEK, polyetheretherketone; PPE, polyethylene; SI, silicone; ME, mersilene.

## Discussion

This systematic review examines alloplastic materials used in facial reconstructive and aesthetic surgery over the past 54 years, including data from 4,279 patients and 13 different materials. Consistent with prior publications, most outcomes reported over the last 54 years focus on PPE, titanium, PEEK, and silicone (3, 5, 128–130). Recent years have seen more reports on PEEK and PPE, while those on silicone, HTR, and ePTFE have decreased (3, 5). PEEK implants have been used to reconstruct complex bony defects, likely due to their intrinsic mechanical stability (8). The increased use of PEEK may be attributed to the ease of handling, improved availability and cost-effectiveness of 3D printing technology for this material in recent years. PPE was the most commonly used material for nearly all facial regions, except the lower face where silicone predominated. However, recent publication trends indicate that PPE has been the most studied material in recent years, while reports on silicone implants have decreased. This suggests a shift in preference from silicone to PPE. Additionally, the increased use of hydroxyapatite-based materials points to a trend toward more natural and biocompatible options.

In this context, the reporting of outcomes differed between studies, and the duration of follow-up varied significantly (5, 131, 132). Notably, 42% of articles did not report any complication in this complex population, with an average follow-up of 25 months. Those studies with longer follow-ups (38 months) noted any complication. Patient-reported outcomes vary significantly between studies, making it challenging to draw definitive conclusions. Despite these variations and limitations, we present fundamental outcome metrics for all implant materials and anatomical locations, which could inform and guide future research.

### Aesthetic and functional outcomes

A significant portion of the studies (44%) reported positive aesthetic results, predominantly with PEEK, titanium, and polyethylene implants. However, the lack of standardized outcome parameters poses a challenge in comparing results across studies. The use of scales such as an aesthetic outcome score or analog visual scales (VAS) in a few studies suggests potential pathways for standardization (Supplementary Table 4). While a substantial number of studies indicated favorable aesthetic results, 16% reported poor outcomes, primarily associated with polyethylene implants. This disparity underscores the need for more consistent reporting and possibly the exploration of other factors influencing aesthetic satisfaction. In terms of functional outcomes, 23% of studies reported improvements, particularly in airway function. However, a notable subset identified ocular issues, such as diplopia and enophthalmos.

### Implant performance variability

No single material consistently outperformed other materials across multiple criteria such as biocompatibility, safety profile, and patient satisfaction. This finding aligns with prior reports on complication rates for different alloplastic materials (3, 5). An example is porous polyethylene (MedPor), which is widely used due to its simplicity, ease of handling, and low complication rate (87, 133). Our review corroborates the generally safe risk-profile of PPE for numerous facial augmentation and reconstruction procedures.

Titanium and PEEK implants are used when structural rigidity is needed (1, 133–135). Other materials, such as hydroxyapatite-based implants, are increasingly used (27, 35). The choice of material seems to be contingent on surgical goals, anatomical considerations; patient wishes, which underscores the need for patient-individualized treatment planning. The highest rate of complication-free postoperative recovery was observed with polyethylene facial implants (FIs). Of the 33 studies on polyethylene, 18 (55%) reported complication-free outcomes, highlighting the favorable safety profile of PPE.

The overall complication rate in the upper face was low, with PPE showing a 13% complication rate, primarily due to implant exposure. Hydroxyapatite-based implants showed no complications among 17 patients. For orbital reconstruction in the midface, PPE, titanium, and hydroxyapatite were the most frequently used implants. This area had higher complication rates, with titanium at 23%, PPE at 13%, hydroxyapatite at 11%, and silicone implants (SI) at 10%. The main complications involved nerve injuries and implant dysfunctions, reflecting the complexity of these cases of orbital reconstruction.

In the non-orbital midface, ePTFE (*n* = 397) was most used often in the setting of rhinoplasty for dorsal augmentation, followed by PPE (*n* = 355) and silicone (*n* = 242). Glass ceramics had the highest complication rate at 23%, while silicone implants had an 8.3% rate. In the lower face, silicone and PPE were the most reported materials, with the region showing the highest overall complication rates: 17% for PPE implants and a substantial 39% for Proplast implants. Many complications were due to hematoma (17% for PPE) and wound dehiscence (19% for Proplast). Silicone implants had a 7.8% complication rate, with bone resorption/erosion reported in about 6% of cases, a complication only noted for silicone in the lower facial region.

### Complications excluding case reports

We analyzed the range of complication rates per implant type in larger studies, explicitly excluding data from case reports and series (Table 4). Using this methodology, unusual or particularly challenging cases, often highlighted in case reports, are omitted. In the previous literature, conclusions are largely based on the summary of case reports which may negatively skew complication rates.

PEEK implants demonstrated the highest complication rate of up to 50% (5 out of 10), followed closely by PPE with up to 48% complication rate (46 out of 95) and Titanium at 35% (9 out of 26). Infection rates varied, with a maximum of 10% reported for ePTFE implants and the lowest at 1% for ME implants. Titanium, PPE, and Silicone showed infection rates up to 5.9%, 7.7%, and 8%, respectively. Wound dehiscence occurred in up to 12% of cases with PPE implants and 20% with GC Proplast. Displacement was most frequent in the Silicone group, reaching up to 14%. Additionally, nerve injuries were most associated with PPE, occurring in 48% of cases.

None of the studies reported on implant related malignancy. In other types of alloplastic implants, specifically macro-textured breast implants, there is a theoretical risk of developing breast implant associated anaplastic large cell lymphoma (BIA-ALCL) (101). This form of malignancy is a type of T-cell non-Hodgkin lymphoma which can develop around an implant within the capsule and is thought to be induced by the implant texture leading to chronic inflammation (81). It is however recognized that this pathologic entity can occur in any part of the body where an implant with a rough surface is implanted (rough implant associated anaplastic large cell lymphoma (RIA-ALCL) (9). For example, the occurrence of ALCL has recently been reported in case of a gluteal implant (81). Although this type of malignancy has not been reported with the use of facial implants, it is a possibility that such an outcome might come to light in the future. Other malignancies such as implant associated squamous cell carcinoma have been associated with various types of implants, including subperiosteal implant of the maxilla and is simply thought to be related to chronic inflammation and stress to squamous epithelium (54).

### Technological advancements

In recent years, the use of patient individualized implants (PSI) has become increasingly popular (136, 137). However, there is a lack of comprehensive studies evaluating their actual benefits in facial implantology and compare it to OTS implants. Current evidence does not consistently demonstrate a significant advantage of PSIs over OTS implants in the upper face and non-orbital midface regions. In orbital midface cases, some studies have reported fewer complications with PSIs compared to OTS implants, which may suggest that customization offers advantages in complex reconstructions, such as orbital repairs (138, 139). However, these findings require further validation. Notably, no infections were reported for PSIs across all regions analyzed, and there were no documented cases of nerve injuries or wound dehiscence, while hematomas were rarely seen. While these findings may indicate potential benefits related to improved surgical planning, shorter operative times, and a better anatomical fit, they should be interpreted with caution. The absence of reported complications does not necessarily imply superiority, as reporting biases, study heterogeneity, and lack of randomized controlled trials limit definitive conclusions. Further prospective studies are necessary to rigorously assess whether PSI implants provide measurable clinical advantages over OTS implants in terms of safety and long-term outcomes.

### Limitations

This systematic review has inherent limitations. The included studies exhibit heterogeneity in outcome reporting, including differences in study design, patient populations, implant types, surgical techniques, and follow-up durations, making direct comparisons challenging and precluding a meta-analysis. Furthermore, publication bias must be considered as positive outcomes are more likely to be submitted for publication. Similarly, in some cases, one must assume underreporting of complications and inconsistencies in complication definitions which introduce data inconsistencies. Lastly, the lack of randomized controlled trials limits the ability to establish causality and the effectiveness of different implant types.

## Conclusion

This systematic review offers a comprehensive analysis of alloplastic materials used in facial reconstructive and aesthetic surgery over the past 54 years. It stresses the need for personalized treatment planning and highlights the need for additional research to better understand each material's safety and efficacy. The review advocates for standardized outcome reporting to enhance comparability and guide future clinical practices. Although some studies suggest that PSIs may help reduce complications such as infections and nerve injuries, the current evidence remains limited. Customization to patient anatomy may offer potential advantages, but further long-term investigations are required to assess the durability, complication rates, and overall clinical impact of these implants. Continued research will be essential in guiding evidence-based treatment decisions.

## Data Availability

The original contributions presented in the study are included in the article/Supplementary Material, further inquiries can be directed to the corresponding authors.
