# Supplementary material for: A systematic review of implant materials for facial reconstructive and aesthetic surgery
Source: Front Surg. 2025 Mar 28;12:1548597. doi: 10.3389/fsurg.2025.1548597 (PMC11985522; doi:10.3389/fsurg.2025.1548597)
Supplement: Supplementary file 1 [file Table1.docx]

**Supplementary Digital Content relevant to the article: A Systematic Review of Implant Materials for Facial Reconstructive and Aesthetic Surgery**

**Systematic Literature Search**

We performed a systematic review by screening the PubMed/MEDLINE, Web of Science, Google Scholar, and EMBASE databases up to January 7, 2024. A search term including four elements was built. Each of the elements was connected by “AND“. The search elements were (i) “alloplastic materials” OR “titanium” OR “silicone” OR “silastic” OR “ceramics” OR “polyethylene” OR “medpor” OR “polymer” OR “polysiloxane” OR “polytetrafluoroethylene” OR “gore-tex” OR “PTFE” OR “mersilene mesh” OR “proplast” OR “polymethylmethacrylate” OR “PMMA” OR “hydroxyapatite” OR “hydroxylapatite” OR “HaP” OR “HA” OR “methacryloyl” OR “polyetheretherketone” OR “PEEK” AND (ii) face” OR “facial” OR “zygoma” OR “frontal” OR “glabella” OR “malar” OR “submalar” OR “maxilla” OR “mandible” OR “mandibular” OR “mandibular angle” OR “jaw” OR “chin” OR “temporal” OR “orbital” AND (iii) “implant” OR “implantology” AND (iv) “reconstruction” OR “reconstructive” OR “contouring” OR “augmentation”. The search string was adapted to the specific syntax of the specific database (Supplement digital content 1). We used the following filters: Case Reports, Classical Article, Clinical Study, Clinical Trial, Clinical Trial, Phase I, Clinical Trial, Phase II, Clinical Trial, Phase III, Clinical Trial, Phase IV, Comparative Study, Meta-Analysis, Multicenter Study, Observational Study, Randomized Controlled Trial. Literature research was conducted from January 7, 2024, to January 14, 2024. Articles included had to be available as full-text and written in English, while animal trials, cadaver, and non-surgical studies were excluded. In cases of multiple studies using the same cohort, we selected studies with the longest follow-up durations. We included all studies that used an alloplastic material to replace missing parts of the craniofacial skeleton (reconstruction) or augment parts of the craniofacial skeleton for aesthetic purposes. Other alloplastic materials used for skeletal fixation (e.g., titanium plates) were excluded.

Two reviewers (L.K. and H.B.) independently screened the articles' titles and abstracts. A subsequent full-text review was performed manually for abstracts considered eligible. Any disagreements were discussed with another reviewer (M.K.-N.) and resolved by consensus.

**Supplementary Table 1**. Search strings in PubMed/MEDLINE, Web of Science, EMBASE, and CENTRAL databases with the respective number of search results.

| Database | Search String | Results |
| --- | --- | --- |
| PubMed/MEDLINE | ("alloplastic materials" OR "titanium" OR "silicone" OR "silastic" OR "ceramics" OR "polyethylene" OR "medpor" OR "polymer" OR "polysiloxane" OR "polytetrafluoroethylene" OR "gore-tex" OR "PTFE" OR "mersilene mesh" OR "proplast" OR "polymethylmethacrylate" OR "PMMA" OR "hydroxyapatite" OR "hydroxylapatite" OR "HaP" OR "HA" OR "methacryloyl" OR "polyetheretherketone" OR "PEEK")  AND  ("face" OR "facial" OR "zygoma" OR "frontal" OR "glabella" OR "malar" OR "submalar" OR "maxilla" OR "mandible" OR "mandibular" OR "mandibular angle" OR "jaw" OR "chin" OR "temporal" OR "orbital")  AND  ("implant" OR "implantology")  AND  ("reconstruction" OR "reconstructive" OR "contouring" OR "augmentation") | 2,315 |
| Web of Science | TS=("alloplastic materials" OR "titanium" OR "silicone" OR "silastic" OR "ceramics" OR "polyethylene" OR "medpor" OR "polymer" OR "polysiloxane" OR "polytetrafluoroethylene" OR "gore-tex" OR "PTFE" OR "mersilene mesh" OR "proplast" OR "polymethylmethacrylate" OR "PMMA" OR "hydroxyapatite" OR "hydroxylapatite" OR "HaP" OR "HA" OR "methacryloyl" OR "polyetheretherketone" OR "PEEK")  AND  TS=("face" OR "facial" OR "zygoma" OR "frontal" OR "glabella" OR "malar" OR "submalar" OR "maxilla" OR "mandible" OR "mandibular" OR "mandibular angle" OR "jaw" OR "chin" OR "temporal" OR "orbital")  AND  TS=("implant" OR "implantology")  AND  TS=("reconstruction" OR "reconstructive" OR "contouring" OR "augmentation") | 2,053 |
| EMBASE | ('alloplastic materials'/exp OR 'titanium'/exp OR 'silicone'/exp OR 'silastic'/exp OR 'ceramics'/exp OR 'polyethylene'/exp OR 'medpor'/exp OR 'polymer'/exp OR 'polysiloxane'/exp OR 'polytetrafluoroethylene'/exp OR 'gore-tex'/exp OR 'PTFE'/exp OR 'mersilene mesh'/exp OR 'proplast'/exp OR 'polymethylmethacrylate'/exp OR 'PMMA'/exp OR 'hydroxyapatite'/exp OR 'hydroxylapatite'/exp OR 'HaP'/exp OR 'HA'/exp OR 'methacryloyl'/exp OR 'polyetheretherketone'/exp OR 'PEEK'/exp)  AND  ('face'/exp OR 'facial'/exp OR 'zygoma'/exp OR 'frontal'/exp OR 'glabella'/exp OR 'malar'/exp OR 'submalar'/exp OR 'maxilla'/exp OR 'mandible'/exp OR | 1,549 |
| CENTRAL | (("alloplastic materials" OR "titanium" OR "silicone" OR "silastic" OR "ceramics" OR "polyethylene" OR "medpor" OR "polymer" OR "polysiloxane" OR "polytetrafluoroethylene" OR "gore-tex" OR "PTFE" OR "mersilene mesh" OR "proplast" OR "polymethylmethacrylate" OR "PMMA" OR "hydroxyapatite" OR "hydroxylapatite" OR "HaP" OR "HA" OR "methacryloyl" OR "polyetheretherketone" OR "PEEK")  AND  ("face" OR "facial" OR "zygoma" OR "frontal" OR "glabella" OR "malar" OR "submalar" OR "maxilla" OR "mandible" OR "mandibular" OR "mandibular angle" OR "jaw" OR "chin" OR "temporal" OR "orbital")  AND  ("implant" OR "implantology")  AND  ("reconstruction" OR "reconstructive" OR "contouring" OR "augmentation")) | 2,122 |

**Supplementary Table 2.** Newcastle Ottawa Score (NOS).

| **Study** | **Selection** | **Comparability** | **Assessment** | **Newcastle-Ottawa Scale** |
| --- | --- | --- | --- | --- |
| Saporano, 2023 ^1^ | *** | ** | *** | 8 |
| Nocini, 2022 ^2^ |  |  |  | 0 |
| Shi, 2022 ^3^ | *** |  | ** | 5 |
| Lim, 2022 ^4^ | *** | * | *** | 7 |
| Ha, 2022 ^5^ |  |  |  | 0 |
| Mayo, 2022 ^6^ | ** |  | ** | 4 |
| Kim, 2022 ^7^ | ** |  | * | 3 |
| Hamsho, 2022 ^8^ |  |  | ** | 2 |
| Darwich, 2021 ^9^ | * |  | ** | 3 |
| Watanabe, 2021 ^10^ | *** | ** | *** | 8 |
| Ramieri, 2021 ^11^ | ** |  | ** | 4 |
| Antúnez-Conde, 2021 ^12^ | *** | ** | ** | 7 |
| Olate, 2021 ^13^ | ** |  | ** | 4 |
| Sesqué, 2021 ^14^ |  |  |  | 0 |
| Yashin, 2021 ^15^ | ** |  | ** | 4 |
| Khashaba, 2021 ^16^ | ** |  | ** | 4 |
| Narciso, 2021 ^17^ | ** |  | ** | 4 |
| Yang, 2021 ^18^ | ** |  | ** | 4 |
| Bai, 2020 ^19^ | **** | ** | *** | 9 |
| Jang, 2020 ^20^ | **** | ** | *** | 9 |
| Mrad, 2020 ^21^ | ** |  | ** | 4 |
| Doh, 2019 ^22^ | ** |  | ** | 4 |
| Scofield-Kaplan, 2019 ^23^ | *** |  | ** | 5 |
| Tsumiyama, 2019 ^24^ | * |  | ** | 3 |
| Suh, 2018 ^25^ | * |  | ** | 3 |
| Woo, 2018 ^26^ | ** |  | * | 3 |
| Findikcioglu, 2018 ^27^ | *** |  | ** | 5 |
| Kanazawa, 2018 ^28^ | ** |  | ** | 4 |
| Al-Jandan, 2018 ^29^ | *** | * | *** | 7 |
| Sciaraffia, 2018 ^30^ | ** |  | ** | 4 |
| Kohyama, 2018 ^31^ | *** |  | *** | 6 |
| Franco, 2017 ^32^ | ** |  | * | 3 |
| [Zieliński](https://pubmed.ncbi.nlm.nih.gov/?term=Zieli%C5%84ski+R&cauthor_id=28318927), 2017 ^33^ | *** |  | ** | 5 |
| Callahan, 2017 ^34^ | ** |  | ** | 4 |
| Sainsbury, 2017 ^35^ | ** |  | ** | 4 |
| Cho, 2017 ^36^ | ** |  | ** | 4 |
| Lee, 2017 ^37^ |  |  |  | 0 |
| Ghosh, 2017 ^38^ | ** |  | ** | 4 |
| Kanno, 2017 ^39^ | *** |  | ** | 5 |
| Hosseini, 2016 ^40^ | ** |  | ** | 4 |
| Joo, 2016 ^41^ | *** | * | *** | 7 |
| Timoney, 2016 ^42^ | ** |  | ** | 4 |
| Hussain, 2016 ^43^ | ** |  | ** | 4 |
| da Silva de Menezes, 2016 ^44^ | ** |  | ** | 4 |
| Park, 2016 ^45^ | ** |  | ** | 4 |
| Polo, 2016 ^46^ | ** |  | ** | 4 |
| Lavie, 2015 ^47^ | *** | * | *** | 7 |
| Nahumi, 2015 ^48^ | * |  | ** | 3 |
| Gander, 2015 ^49^ | ** | * | ** | 5 |
| Yim, 2015 ^50^ | *** |  | *** | 6 |
| Rotaru, 2015 ^51^ | ** |  | ** | 4 |
| Park, 2015 ^52^ | *** |  | ** | 5 |
| Jalbert, 2014 ^53^ | ** |  | ** | 4 |
| Atherton, 2014 ^54^ | ** |  | ** | 4 |
| Kozakiewicz, 2013 ^55^ | *** | ** | *** | 8 |
| Kozakiewicz, 2013 ^56^ | *** | ** | *** | 8 |
| Alonso, 2013 ^57^ |  |  |  | 0 |
| Hatamleh, 2013 ^58^ | ** |  | ** | 4 |
| Hayashi, 2013 ^59^ | ** | * | ** | 5 |
| Guo, 2012 ^60^ | *** | ** | *** | 8 |
| Kim, 2012 ^61^ | ** | * | *** | 6 |
| Scolozzi, 2012 ^62^ |  |  |  | 0 |
| Niechajev, 2012 ^63^ | *** | ** | *** | 8 |
| Lin, 2012 ^64^ | *** | * | *** | 7 |
| Kirby, 2011 ^65^ | *** | ** | *** | 8 |
| Kim, 2011 ^66^ | *** | ** | *** | 8 |
| Aynehchi, 2011 ^67^ | **** | ** | *** | 9 |
| Atherton, 2010 ^68^ | *** | ** | *** | 8 |
| Park, 2010 ^69^ | *** | * | *** | 7 |
| Li, 2010 ^70^ | *** | ** | *** | 8 |
| Chen, 2010 ^71^ | *** |  | *** | 6 |
| Tang, 2010 ^72^ | *** | * | *** | 7 |
| Hopping, 2010 ^73^ | *** | ** | *** | 8 |
| Deshpande, 2010 ^74^ | *** | ** | *** | 8 |
| Kim, 2009 ^75^ | *** | * | ** | 7 |
| Stringer, 2009 ^76^ | *** | * | *** | 7 |
| Jirman, 2009 ^77^ | *** |  | *** | 6 |
| Guo, 2009 ^78^ | *** | ** | *** | 8 |
| Emsen, 2008 ^79^ | *** | ** | ** | 7 |
| Gui, 2008 ^80^ | *** | * | *** | 7 |
| Coban, 2008 ^81^ | *** | * | *** | 7 |
| Garibaldi, 2007 ^82^ | *** | ** | *** | 8 |
| Scholz, 2007 ^83^ | *** | ** | *** | 8 |
| Eski, 2007 ^84^ | *** | ** | *** | 8 |
| Ozturk, 2006 ^85^ | *** | * | *** | 7 |
| Romo, 2006 ^86^ | *** | * | *** | 7 |
| Gürlek, 2006 ^87^ | *** | ** | *** | 8 |
| Thornton, 2006 ^88^ | *** | ** | *** | 8 |
| Menderes, 2004 ^89^ | *** | ** | ** | 7 |
| Ellis, 2003 ^90^ | *** | ** | ** | 7 |
| Yaremchuck, 2003 ^91^ | *** | ** | *** | 8 |
| Dusková, 2002 ^92^ | *** | ** | *** | 8 |
| Saleh, 2002 ^93^ | *** | * | *** | 7 |
| Lustica, 2001 ^94^ |  |  |  | 0 |
| Sevin, 2000 ^95^ | *** | * | *** | 7 |
| Ramirez, 2000 ^96^ | *** | ** | *** | 8 |
| Yaremchuck, 2000 ^97^ | *** | * | *** | 7 |
| Fedok, 1999 ^98^ |  |  |  | 0 |
| Metzinger, 1999 ^99^ | *** | ** | *** | 8 |
| Mendelsohn, 1998 ^100^ | *** |  | *** | 6 |
| Karras, 1998 ^101^ | ** | ** | *** | 7 |
| Yaremchuk, 1998 ^102^ | *** | ** | *** | 8 |
| Frodel, 1998 ^103^ | *** | * | *** | 7 |
| Hirano, 1997 ^104^ | *** | * | *** | 7 |
| Abrahams, 1997 ^105^ | *** |  | *** | 6 |
| Semergidis, 1996 ^106^ | *** |  | *** | 6 |
| Vuyk, 1996 ^107^ | *** | * | *** | 7 |
| Eppley, 1995 ^108^ | *** | * | *** | 7 |
| Matarasso, 1995 ^109^ | *** | * | *** | 7 |
| Owsley, 1994 ^110^ | *** | * | *** | 7 |
| [Ono](https://pubmed.ncbi.nlm.nih.gov/?term=Ono+I&cauthor_id=7831549), 1994 ^111^ | *** | * | *** | 7 |
| Blake, 1990 ^112^ | *** | * | *** | 7 |
| Moenning, 1989 ^113^ | *** |  | *** | 6 |
| Epker, 1989 ^114^ | *** |  | *** | 6 |
| Pitanguy, 1986 ^115^ | *** |  | ** | 5 |
| Dann, 1977 ^116^ | ** |  | ** | 4 |
| Laub, 1970 ^117^ |  |  |  | 0 |

**Supplementary Table 3.** Level of Evidence (LOE).

| **Study** | **Level of Evidence** |
| --- | --- |
| Saporano, 2023 ^1^ | IV |
| Nocini, 2022 ^2^ | IV |
| Shi, 2022 ^3^ | IV |
| Lim, 2022 ^4^ | IV |
| Ha, 2022 ^5^ | IV |
| Mayo, 2022 ^6^ | IV |
| Kim, 2022 ^7^ | IV |
| Hamsho, 2022 ^8^ | IV |
| Darwich, 2021 ^9^ | IV |
| Watanabe, 2021 ^10^ | IV |
| Ramieri, 2021 ^11^ | IV |
| Antúnez-Conde, 2021 ^12^ | IV |
| Olate, 2021 ^13^ | IV |
| Sesqué, 2021 ^14^ | IV |
| Yashin, 2021 ^15^ | IV |
| Khashaba, 2021 ^16^ | IV |
| Narciso, 2021 ^17^ | IV |
| Yang, 2021 ^18^ | IV |
| Bai, 2020 ^19^ | II |
| Jang, 2020 ^20^ | III |
| Mrad, 2020 ^21^ | IV |
| Doh, 2019 ^22^ | IV |
| Scofield-Kaplan, 2019 ^23^ | IV |
| Tsumiyama, 2019 ^24^ | III |
| Suh, 2018 ^25^ | VI |
| Woo, 2018 ^26^ | IV |
| Findikcioglu, 2018 ^27^ | IV |
| Kanazawa, 2018 ^28^ | IV |
| Al-Jandan, 2018 ^29^ | III |
| Sciaraffia, 2018 ^30^ | III |
| Kohyama, 2018 ^31^ | III |
| Franco, 2017 ^32^ | IV |
| [Zieliński](https://pubmed.ncbi.nlm.nih.gov/?term=Zieli%C5%84ski+R&cauthor_id=28318927), 2017 ^33^ | III |
| Callahan, 2017 ^34^ | IV |
| Sainsbury, 2017 ^35^ | IV |
| Cho, 2017 ^36^ | IV |
| Lee, 2017 ^37^ | IV |
| Ghosh, 2017 ^38^ | IV |
| Kanno, 2017 ^39^ | IV |
| Hosseini, 2016 ^40^ | IV |
| Joo, 2016 ^41^ | III |
| Timoney, 2016 ^42^ | IV |
| Hussain, 2016 ^43^ | IV |
| da Silva de Menezes, 2016 ^44^ | IV |
| Park, 2016 ^45^ | IV |
| Polo, 2016 ^46^ | IV |
| Lavie, 2015 ^47^ | IV |
| Nahumi, 2015 ^48^ | IV |
| Gander, 2015 ^49^ | IV |
| Yim, 2015 ^50^ | IV |
| Rotaru, 2015 ^51^ | IV |
| Park, 2015 ^52^ | IV |
| Jalbert, 2014 ^53^ | IV |
| Atherton, 2014 ^54^ | IV |
| Kozakiewicz, 2013 ^55^ | II |
| Kozakiewicz, 2013 ^56^ | IV |
| Alonso, 2013 ^57^ | IV |
| Hatamleh, 2013 ^58^ | IV |
| Hayashi, 2013 ^59^ | IV |
| Guo, 2012 ^60^ | III |
| Kim, 2012 ^61^ | IV |
| Scolozzi, 2012 ^62^ | IV |
| Niechajev, 2012 ^63^ | IV |
| Lin, 2012 ^64^ | IV |
| Kirby, 2011 ^65^ | IV |
| Kim, 2011 ^66^ | IV |
| Aynehchi, 2011 ^67^ | IV |
| Atherton, 2010 ^68^ | IV |
| Park, 2010 ^69^ | IV |
| Li, 2010 ^70^ | IV |
| Chen, 2010 ^71^ | IV |
| Tang, 2010 ^72^ | III |
| Hopping, 2010 ^73^ | III |
| Deshpande, 2010 ^74^ | IV |
| Kim, 2009 ^75^ | IV |
| Stringer, 2009 ^76^ | IV |
| Jirman, 2009 ^77^ | IV |
| Guo, 2009 ^78^ | III |
| Emsen, 2008 ^79^ | IV |
| Gui, 2008 ^80^ | III |
| Coban, 2008 ^81^ | IV |
| Garibaldi, 2007 ^82^ | IV |
| Scholz, 2007 ^83^ | IV |
| Eski, 2007 ^84^ | IV |
| Ozturk, 2006 ^85^ | IV |
| Romo, 2006 ^86^ | IV |
| Gürlek, 2006 ^87^ | IV |
| Thornton, 2006 ^88^ | IV |
| Menderes, 2004 ^89^ | IV |
| Ellis, 2003 ^90^ | III |
| Yaremchuck, 2003 ^91^ | IV |
| Dusková, 2002 ^92^ | IV |
| Saleh, 2002 ^93^ | IV |
| Lustica, 2001 ^94^ | IV |
| Sevin, 2000 ^95^ | IV |
| Ramirez, 2000 ^96^ | IV |
| Yaremchuck, 2000 ^97^ | IV |
| Fedok, 1999 ^98^ | IV |
| Metzinger, 1999 ^99^ | IV |
| Mendelsohn, 1998 ^100^ | IV |
| Karras, 1998 ^101^ | IV |
| Yaremchuk, 1998 ^102^ | IV |
| Frodel, 1998 ^103^ | IV |
| Hirano, 1997 ^104^ | IV |
| Abrahams, 1997 ^105^ | IV |
| Semergidis, 1996 ^106^ | IV |
| Vuyk, 1996 ^107^ | IV |
| Eppley, 1995 ^108^ | III |
| Matarasso, 1995 ^109^ | IV |
| Owsley, 1994 ^110^ | IV |
| [Ono](https://pubmed.ncbi.nlm.nih.gov/?term=Ono+I&cauthor_id=7831549), 1994 ^111^ | IV |
| Blake, 1990 ^112^ | IV |
| Moenning, 1989 ^113^ | III |
| Epker, 1989 ^114^ | IV |
| Pitanguy, 1986 ^115^ | IV |
| Dann, 1977 ^116^ | IV |
| Laub, 1970 ^117^ | IV |

**Supplementary Table 4.** Aesthetic outcomes in various implant materials.

| **Implant Material** | | | | | | | | | |
| --- | --- | --- | --- | --- | --- | --- | --- | --- | --- |
| **TI** | **PEEK** | **PPE** | **ME** | **HA(/PLLA)** | **SI** | **PMMA** | **ePTFE** | **HTR** | **PCL** |
| 8.69 ± 1.01 (VAS; visual analog scale) ^4^ | "good symmetrisation" ^2^ | "no midface flattening, good position of the lower eyelid, and no enophthalmos" ^23^ | "pleasing cosmetic outcomes" ^68^ | anatomical accuracy of the implant placement (postoperatice CT images): excellent: 90% (54/60) (PLC), 73% (44/60) (HA), poor: 10% (6/60) (PLC), 27% (16/60) (HA) ^20^ | "aesthetic bonus" ^21^ | 5.3% (1/19) (sagging of the borders of the implant) ^94^ | aesthetic outcome score: 2.99 (score: 1-4) (comparable to the costal cartilage group, p=0.93) ^41^ | "providing the desired aesthetic change, without causing resorption of underlying bone" ^101^ | anatomical accuracy of the implant placement (postoperatice CT images): excellent: 90% (54/60) (PLC), 73% (44/60) (HA), poor: 10% (6/60) (PLC), 27% (16/60) (HA) ^20^ |
| "perfect results at the level of facial symmetry" ^9^ | "patient satisfaction with cosmetic result" ^3^ | 1.5-mm enophthalmos, upper and lower punctal obstruction, scarring (left eye) ^25^ |  | "excellent cosmetic appearene" ^104^ | "custom silicone implants were advantageous in predicting treatment outcomes", "well placed" ^50^ |  | "good restoration of symmetry" ^98^ |  |  |
| "optimal aesthetic results" ^12^ | "improvement of facial esthetics" ^5^ | "good restoration of the globe position", "correction of enophthalmos" ^34^ |  |  | "satisfactory aesthetic results" ^67^ |  |  |  |  |
| "symmetrical and satisfactory facial morphology" ^14^ | "the result was in line with the set goals" ^11^ | residual lower lid retraction, lateral pull at the lateral canthus ^32^ |  |  | MAL: 2.0% (2/100) (asymmetry) ^73^ |  |  |  |  |
| "good" | "esthetic acceptance" ^16^ | "aestetic result", "minimal scar" ^40^ |  |  | VAS (0:poor, 10:excellent): mean 2 (range, 1-4) ^99^ |  |  |  |  |
| "successfully restored contour of overresected mandible to the original status" ^15^ | "aesthetic improvement" ^17^ | "correction of facial harmony", "balance of the facial thirds, paranasal projection" ^44^ |  |  | poor cosmetic results: (1/4) 25% ^105^ |  |  |  |  |
| 17% (2/12) (overextent of implant) ^26^ | "good appearance, excellent restoration of contour and esthetics" ^18^ | aesthetic failure (4/90) (4.4%) ^63^ |  |  | 7.5% (3/40) (asymmetry) ^107^ |  |  |  |  |
| "residual asymmetry" ^49^ | "symmetric" ^22^ | good results: 95% (90/95), chin "too strong": 4.2% (4/95), poor transition 1.1% (1/95) ^64^ |  |  | "optimal esthetics" ^109^ |  |  |  |  |
| "fitting accurately" ^51^ | "pleasing aestetic result" ^35^ | mean relapse rate of Pogs: 15% (augmentetion with osteotomy: 19%) ^69^ |  |  | "ranged from good to excellent" ^114^ |  |  |  |  |
| "facial malformation were improved" ^58^ | "good contour and volume of the left midfacial region" ^43^ | esthetic-improvement rate: 91% (29/32) ^71^ |  |  | 4.0% (1/25) (prothesis slighly large) ^117^ |  |  |  |  |
| "satisfactory aesthetic outcomes" ^76^ | "excellent cosmetic result" ^47^ | "maintaining a natural appearance and feel ^74^" |  |  |  |  |  |  |  |
| "good" ^83^ | left-sided temporal hollowing deformity ^48^ | "limited extent of resultant scars" ^79^ |  |  |  |  |  |  |  |
| "pleasing cosmetic result" ^112^ | osirix software and adobe photoshop: analysis of the position between the bone resection margins and the implant and the implant projection compared to the mirrored opposite side: excellent cosmetic results ^53^ | "satisfactory results" ^80^ |  |  |  |  |  |  |  |
| "no midface flattening, good position of the lower eyelid, and no enophthalmos" ^23^ | "stable cosmetic reconstruction" ^62^ | "perfect contour and symmetry " ^84^ |  |  |  |  |  |  |  |
| "good restoration of the globe position", "correction of enophthalmos" ^34^ | "improved postoperative bony and soft-tissue facial contours", "decreased enophthalmos" ^75^ | improvement of facial symmetry: lipofilling of the cheek nasal junction ^85^ |  |  |  |  |  |  |  |

TI, titanium; PEEK, polyetheretherketone; PPE, polyethylene; ePTFE, expanded polytetrafluorethylene; SI, silicone; ME, mersilene; HA/PLLA, hydroxylapatite/poly-l-lactide; PCL, polycaprolactone; PMMA, polymethylmethacrylate; GC, glass ceramics; PRO, Proplast; ZR, zirconium oxide; HTR, hard tissue replacement.

References

1. Saponaro G, Todaro M, Barbera G, et al. Patient-Specific Facial Implants in Polyetheretherketone and Their Stability: A Preliminary Study. Annals of Plastic Surgery 2023: 90: 564-67.

2. Nocini R, D'Agostino A, Trevisiol L, Favero V. Mandibular recontouring with polyetheretherketone (PEEK) patient-specific implants. BMJ Case Rep 2022: 15.

3. Shi H, Yin X, Hu Y. Solitary Neurofibroma of the Zygoma: Three-Dimensional Virtual Resection and Patient-Specific Polyetheretherketone Implant Reconstruction. J Craniofac Surg 2022: 33: e781-e83.

4. Lim HK, Choi YJ, Choi WC, Song IS, Lee UL. Reconstruction of maxillofacial bone defects using patient-specific long-lasting titanium implants. Sci Rep 2022: 12: 7538.

5. Ha SH, Lee H, Choi JY. Correction of Midface Deficiency in Patient With Crouzon Syndrome by Orthognathic Surgery and Patient Specific Facial Implant: Case Report. J Craniofac Surg 2022: 33: e191-e94.

6. Mayo W, Mohamad AH, Zazo H, et al. Facial defects reconstruction by titanium mesh bending using 3D printing technology: A report of two cases. Annals of Medicine and Surgery 2022: 78: 103837.

7. Kim MW, Kim SH, Nam SB, et al. Reconstruction of temporal hollowing deformities using silicone implants made using a toy-clay model: a report of three cases. Arch Craniofac Surg 2022: 23: 34-38.

8. Hamsho R, Mahardawi B, Assi H, Alkhatib H. Polyetheretherketone (PEEK) Implant for the Reconstruction of Severe Destruction in the Maxilla: Case Report. Plastic and Reconstructive Surgery – Global Open 2022: 10: e4473.

9. Darwich K, Ismail MB, Al-Mozaiek MYA, Alhelwani A. Reconstruction of mandible using a computer-designed 3D-printed patient-specific titanium implant: a case report. Oral Maxillofac Surg 2021: 25: 103-11.

10. Watanabe A, Yamanaka Y, Rajak SN, et al. Assessment of a Consecutive Series of Orbital Floor Fracture Repairs With the Hess Area Ratio and the Use of Unsintered Hydroxyapatite Particles/Poly l-Lactide Composite Sheets for Orbital Fracture Reconstruction. J Oral Maxillofac Surg 2021: 79: 420-28.

11. Ramieri V, Maffìa F, Vellone V, Marianetti S, Marianetti TM. The Pyramid Chin Augmentation: A New Technique. J Craniofac Surg 2021: 32: 738-39.

12. Antúnez-Conde R, Salmerón JI, Navarro C. Intraosseous Venous Malformation of the Zygomatic Bone: Virtual Surgical Planning and Reconstruction With Customized CAD-CAM Titanium Implant. J Craniofac Surg 2021: 32: e757-e59.

13. Olate S, Huetequeo-Molina C, Requena R, Uribe F. Patient Specific Implants to Solve Structural Facial Asymmetry After Orthognathic Surgery. J Craniofac Surg 2021: 32: e269-e71.

14. Sesqué A, Dang NP, Coste A, Barthélémy I, Depeyre A. Orbitofrontal Reconstruction With a Three-Dimensional Titanium Patient-Specific Implant After Intraosseous Haemangioma Resection. J Craniofac Surg 2021: 32: e69-e72.

15. Yashin KS, Ermolaev AY, Ostapyuk MV, et al. Case Report: Simultaneous Resection of Bone Tumor and CAD/CAM Titanium Cranioplasty in Fronto-Orbital Region. Front Surg 2021: 8: 718725.

16. Khashaba MM, Shaheen HA, Ibrahim WH, AlDainy DG. Accuracy of patient-specific temporal implants using PEKK. J Craniomaxillofac Surg 2021: 49: 943-49.

17. Narciso R, Basile E, Bottini DJ, Cervelli V. PEEK Implants: An Innovative Solution for Facial Aesthetic Surgery. Case Rep Surg 2021: 2021: 5518433.

18. Yang M, Wu Z, Yu H, Cheng J. Reconstruction for diverse fronto-orbital defects with computer-assisted designed and computer-assisted manufactured PEEK implants in one-stage operation: Case reports. Medicine (Baltimore) 2021: 100: e27452.

19. Bai SS, Li D, Xu L, et al. A Novel Method to Enhance Dynamic Rhinoplasty Outcomes: Double "V" Carving for Alloplastic Grafts. Ear Nose Throat J 2020: 99: 262-67.

20. Jang HU, Kim SY. Biodegradable implants for orbital wall fracture reconstruction. Arch Craniofac Surg 2020: 21: 99-105.

21. Mrad MA, Shah Mardan QNM, Mahabbat NA. Radicular cysts and Chin implants; An unexpected complication prompting explantation - Case report. Int J Surg Case Rep 2020: 77: 766-68.

22. Doh G, Eo S, Hong KY. Temporal Hollowing Augmentation With Polyetheretherketone Patient-Specific Implant. J Craniofac Surg 2019: 30: 2131-33.

23. Scofield-Kaplan SM, Patel SY, Mancini R. Orbital Floor and Rim Reconstruction With a Titanium Orbital Implant and Acellular Dermis. Ophthalmic Plast Reconstr Surg 2019: 35: e19-e21.

24. Tsumiyama S, Umeda G, Ninomiya K, Miyawaki T. Use of Unsintered Hydroxyapatite and Poly-l-lactic Acid Composite Sheets for Management of Orbital Wall Fracture. Journal of Craniofacial Surgery 2019: 30: 2001-03.

25. Suh SY, Yeom JA, Ahn JH. Porous Polyethylene Implant Associated With Delayed Orbital Complications and Osteomyelitis After Orbital Reconstruction. J Craniofac Surg 2018: 29: 1910-11.

26. Woo JM, Baek SH, Kim JC, Choi JY. Contour Restoration of Over-Resected Mandibular Angle and Lower Border by Reduction Mandibuloplasty Using Three-Dimensional Planning and Computer-Aided Design and Manufacturing Custom-Made Titanium Implants. J Craniofac Surg 2018: 29: e340-e43.

27. Findikcioglu K, Sibar S, Gulsen A. Treatment Approach to Severe Microgenia Cases: Combined Use of Osseous and Implant Genioplasty. J Craniofac Surg 2018: 29: e175-e79.

28. Kanazawa S, Kiya K, Kubo T, Hosokawa K. Hydroxyapatite implantation for the repair of a congenital nasal anomaly: 10 years follow-up. Journal of Surgical Case Reports 2018: 2018.

29. Al-Jandan B, Marei HF. Mandibular angle augmentation using solid silicone implants. Dent Med Probl 2018: 55: 367-70.

30. Sciaraffia CE, Ahumada MF, Parada FJ, Gonzalez E, Prado A. Bone Resorption after Use of Silicone Chin Implants, Long-term Follow-up Study with Lateral Chin Radiography. Plast Reconstr Surg Glob Open 2018: 6: e1850.

31. Kohyama K, Morishima Y, Arisawa K, Arisawa Y, Kato H. Immediate and long-term results of unsintered hydroxyapatite and poly L-lactide composite sheets for orbital wall fracture reconstruction. Journal of Plastic, Reconstructive & Aesthetic Surgery 2018: 71: 1069-75.

32. Franco J, Harris MS, Vernon D, Shipchandler TZ. Reconstruction of midface defect from idiopathic destructive process using Medpor implant. Am J Otolaryngol 2017: 38: 351-53.

33. Zieliński R, Malińska M, Kozakiewicz M. Classical versus custom orbital wall reconstruction: Selected factors regarding surgery and hospitalization. J Craniomaxillofac Surg 2017: 45: 710-15.

34. Callahan AB, Campbell AA, Petris C, Kazim M. Low-Cost 3D Printing Orbital Implant Templates in Secondary Orbital Reconstructions. Ophthalmic Plast Reconstr Surg 2017: 33: 376-80.

35. Sainsbury DC, George A, Forrest CR, Phillips JH. Bilateral Malar Reconstruction Using Patient-Specific Polyether Ether Ketone Implants in Treacher-Collins Syndrome Patients With Absent Zygomas. J Craniofac Surg 2017: 28: 515-17.

36. Cho WK, Ko AC, Korn BS, Kikkawa DO. Orbitocutaneous Fistula Secondary to Buried Polyethylene Mesh Implant 12 Years After Injury. Ophthalmic Plast Reconstr Surg 2017: 33: e107-e08.

37. Lee EI. Aesthetic Alteration of the Chin. Semin Plast Surg 2013: 27: 155-60.

38. Ghosh S, Pramanick D, Ray A, Burman R, Saha A. Fronto-orbital reconstruction using polymethyl methacrylate implant. Natl J Maxillofac Surg 2017: 8: 153-56.

39. Kanno T, Karino M, Yoshino A, et al. Feasibility of Single Folded Unsintered Hydroxyapatite Particles/Poly-L-Lactide Composite Sheet in Combined Orbital Floor and Medial Wall Fracture Reconstruction. Journal of Hard Tissue Biology 2017: 26: 237-44.

40. Hosseini SN, Alizadeh A, Zahedi A. Reconstructing a Giant Frontal Osteoma With Porex. J Craniofac Surg 2016: 27: 2078-80.

41. Joo YH, Jang YJ. Comparison of the Surgical Outcomes of Dorsal Augmentation Using Expanded Polytetrafluoroethylene or Autologous Costal Cartilage. JAMA Facial Plast Surg 2016: 18: 327-32.

42. Timoney PJ, Clark JD, Frederick PA, et al. Foreign Body Granuloma Following Orbital Reconstruction with Porous Polyethylene. Ophthalmic Plast Reconstr Surg 2016: 32: e137-e38.

43. Hussain RN, Clark M, Berry-Brincat A. The Use of a Polyetheretherketone (PEEK) Implant to Reconstruct the Midface Region. Ophthalmic Plast Reconstr Surg 2016: 32: e151-e53.

44. da Silva de Menezes JD, Moura LB, Martins RP, Hochuli-Vieira E. Porous Polyethylene Implant as Aesthetic Complement in Orthognathic Surgery. J Craniofac Surg 2016: 27: e790-e91.

45. Park YW. Frontal augmentation as an adjunct to orthognathic or facial contouring surgery. Maxillofac Plast Reconstr Surg 2016: 38: 37.

46. Polo M. Bone resorption under chin implants: The orthodontist's role in its diagnosis and management. Am J Orthod Dentofacial Orthop 2017: 151: 201-08.

47. Lavie J, Stalder MW, St Hilaire H. Virtual Resection and Subsequent Design of a Patient-Specific Alloplastic Implant in the Preoperative Planning and Surgical Treatment of a Venous Malformation of the Zygoma. J Craniofac Surg 2015: 26: e641-3.

48. Nahumi N, Shohet MR, Bederson JB, Elahi E. Frontorbital Fibrous Dysplasia Resection and Reconstruction With Custom Polyetherlatone Alloplast. J Craniofac Surg 2015: 26: e720-2.

49. Gander T, Essig H, Metzler P, et al. Patient specific implants (PSI) in reconstruction of orbital floor and wall fractures. J Craniomaxillofac Surg 2015: 43: 126-30.

50. Yim HW, Nguyen A, Kim YK. Facial Contouring Surgery with Custom Silicone Implants Based on a 3D Prototype Model and CT-Scan: A Preliminary Study. Aesthetic Plastic Surgery 2015: 39: 418-24.

51. Rotaru H, Schumacher R, Kim SG, Dinu C. Selective laser melted titanium implants: a new technique for the reconstruction of extensive zygomatic complex defects. Maxillofac Plast Reconstr Surg 2015: 37: 1.

52. Park H, Kim HS, Lee BI. Medial Wall Orbital Reconstruction using Unsintered Hydroxyapatite Particles/Poly L-Lactide Composite Implants. Arch Craniofac Surg 2015: 16: 125-30.

53. Jalbert F, Boetto S, Nadon F, et al. One-step primary reconstruction for complex craniofacial resection with PEEK custom-made implants. J Craniomaxillofac Surg 2014: 42: 141-8.

54. Atherton D, Haers P. Midfacial augmentation in teenage cleft patients using malar and paranasal Medpor implants. Int J Oral Maxillofac Surg 2014: 43: 824-6.

55. Kozakiewicz M, Szymor P. Comparison of pre-bent titanium mesh versus polyethylene implants in patient specific orbital reconstructions. Head Face Med 2013: 9: 32.

56. Kozakiewicz M, Elgalal M, Walkowiak B, Stefanczyk L. Technical concept of patient-specific, ultrahigh molecular weight polyethylene orbital wall implant. J Craniomaxillofac Surg 2013: 41: 282-90.

57. Alonso N, de Pochat VD, de Barros AR, Tavares LS. Long-term complication after rhinoplasty using porous polyethylene implant: cutaneous fistula of the forehead. J Craniofac Surg 2013: 24: 2176-8.

58. Hatamleh MM, Cartmill M, Watson J. Management of extensive frontal cranioplasty defects. J Craniofac Surg 2013: 24: 2018-22.

59. Hayashi M, Muramatsu H, Sato M, et al. Surgical treatment of facial fracture by using unsintered hydroxyapatite particles/poly l-lactide composite device (OSTEOTRANS MX®): A clinical study on 17 cases. Journal of Cranio-Maxillofacial Surgery 2013: 41: 783-88.

60. Guo J, Tian W, Long J, et al. A retrospective study of traumatic temporal hollowing and treatment with titanium mesh. Ann Plast Surg 2012: 68: 279-85.

61. Kim CY, Jeong BJ, Lee SY, Yoon JS. Comparison of surgical outcomes of large orbital fractures reconstructed with porous polyethylene channel and porous polyethylene titan barrier implants. Ophthalmic Plast Reconstr Surg 2012: 28: 176-80.

62. Scolozzi P. Maxillofacial reconstruction using polyetheretherketone patient-specific implants by "mirroring" computational planning. Aesthetic Plast Surg 2012: 36: 660-5.

63. Niechajev I. Facial Reconstruction Using Porous High-Density Polyethylene (Medpor): Long-Term Results. Aesthetic Plastic Surgery 2012: 36: 917-27.

64. Lin J, Chen X. Modified Technique of Chin Augmentation With MEDPOR for Asian Patients. Aesthetic Surgery Journal 2012: 32: 799-803.

65. Kirby EJ, Turner JB, Davenport DL, Vasconez HC. Orbital floor fractures: outcomes of reconstruction. Ann Plast Surg 2011: 66: 508-12.

66. Kim YH, Kim TG, Lee JH, Nam HJ, Lim JH. Inlay implanting technique for the correction of medial orbital wall fracture. Plast Reconstr Surg 2011: 127: 321-26.

67. Aynehchi BB, Burstein DH, Parhiscar A, Erlich MA. Vertical incision intraoral silicone chin augmentation. Otolaryngol Head Neck Surg 2012: 146: 553-9.

68. Atherton DD, Joshi N, Kirkpatrick N. Augmentation of temporal fossa hollowing with Mersilene mesh. J Plast Reconstr Aesthet Surg 2010: 63: 1629-34.

69. Park JY, Kim SG, Baik SM, Kim SY. Comparison of genioplasty using Medpor and osteotomy. Oral Surg Oral Med Oral Pathol Oral Radiol Endod 2010: 109: e26-30.

70. Li M, Lin X, Xu Y. The application of rapid prototyping technique in chin augmentation. Aesthetic Plast Surg 2010: 34: 172-8.

71. Chen CT, Hu TL, Lai JB, Chen YC, Chen YR. Reconstruction of traumatic nasal deformity in Orientals. J Plast Reconstr Aesthet Surg 2010: 63: 257-64.

72. Tang W, Guo L, Long J, et al. Individual design and rapid prototyping in reconstruction of orbital wall defects. J Oral Maxillofac Surg 2010: 68: 562-70.

73. Hopping SB, Joshi AS, Tanna N, Janjanin S. Volumetric Facelift: Evaluation of Rhytidectomy with Alloplastic Augmentation. Annals of Otology, Rhinology & Laryngology 2010: 119: 174-80.

74. Deshpande S, Munoli A. Long-term results of high-density porous polyethylene implants in facial skeletal augmentation: An Indian perspective. Indian J Plast Surg 2010: 43: 34-9.

75. Kim MM, Boahene KD, Byrne PJ. Use of customized polyetheretherketone (PEEK) implants in the reconstruction of complex maxillofacial defects. Arch Facial Plast Surg 2009: 11: 53-7.

76. Stringer D, Brown B. Correction of mandibular asymmetry using angled titanium mesh. J Oral Maxillofac Surg 2009: 67: 1619-27.

77. Jirman R, Horák Z, Mazánek J, Reznícek J. Individual replacement of the frontal bone defect: case report. Prague Med Rep 2009: 110: 79-84.

78. Guo L, Tian W, Feng F, et al. Reconstruction of orbital floor fractures: comparison of individual prefabricated titanium implants and calvarial bone grafts. Ann Plast Surg 2009: 63: 624-31.

79. Emsen IM, Benlier E. A new approach on reconstruction of frontonasal encephalomeningocele assisted with medpor. J Craniofac Surg 2008: 19: 537-9.

80. Gui L, Huang L, Zhang Z. Genioplasty and chin augmentation with Medpore implants: a report of 650 cases. Aesthetic Plast Surg 2008: 32: 220-6.

81. Coban YK, Kabalci SK. Surgical treatment of posttraumatic enophthalmos with diced medpor implants through mini-lateral canthoplasty incision. J Craniofac Surg 2008: 19: 539-41.

82. Garibaldi DC, Iliff NT, Grant MP, Merbs SL. Use of porous polyethylene with embedded titanium in orbital reconstruction: a review of 106 patients. Ophthalmic Plast Reconstr Surg 2007: 23: 439-44.

83. Scholz M, Wehmöller M, Lehmbrock J, et al. Reconstruction of the temporal contour for traumatic tissue loss using a CAD/CAM-prefabricated titanium implant-case report. J Craniomaxillofac Surg 2007: 35: 388-92.

84. Eski M, Sengezer M, Turegun M, Deveci M, Isik S. Contour Restoration of the Secondary Deformities of Zygomaticoorbital Fractures With Porous Polyethylene Implant. Journal of Craniofacial Surgery 2007: 18: 520-25.

85. Ozturk S, Acarturk TO, Yapici K, Sengezer M. Treatment of 'en coup de sabre' deformity with porous polyethylene implant. J Craniofac Surg 2006: 17: 696-701.

86. Romo T, 3rd, Kwak ES. Difficult revision case: Overaggressive resection. Facial Plast Surg Clin North Am 2006: 14: 411-5, viii.

87. Gürlek A, Ersoz-Ozturk A, Celik M, et al. Correction of the crooked nose using custom-made high-density porous polyethylene extended spreader grafts. Aesthetic Plast Surg 2006: 30: 141-9.

88. Thornton MA, Mendelsohn M. Total skeletal reconstruction of the nasal dorsum. Arch Otolaryngol Head Neck Surg 2006: 132: 1183-8.

89. Menderes A, Baytekin C, Topcu A, Yilmaz M, Barutcu A. Craniofacial reconstruction with high-density porous polyethylene implants. J Craniofac Surg 2004: 15: 719-24.

90. Ellis E, 3rd, Tan Y. Assessment of internal orbital reconstructions for pure blowout fractures: cranial bone grafts versus titanium mesh. J Oral Maxillofac Surg 2003: 61: 442-53.

91. Yaremchuk MJ. Facial Skeletal Reconstruction Using Porous Polyethylene Implants. Plastic and Reconstructive Surgery 2003: 111: 1818-27.

92. Dusková M, Smahel Z, Vohradník M, et al. Bioactive glass-ceramics in facial skeleton contouring. Aesthetic Plast Surg 2002: 26: 274-83.

93. SALEH HA, LOHUIS PJFM, VUYK HD. Bone resorption after alloplastic augmentation of the mandible. Clinical Otolaryngology & Allied Sciences 2002: 27: 129-32.

94. Lustica I, Velepic M, Cvjetković N, et al. Polymethyl-methacrylate implants in forehead and supraorbital arches reconstruction: retrospective study. Coll Antropol 2001: 25 Suppl: 137-43.

95. Sevin K, Askar I, Saray A, Yormuk E. Exposure of high-density porous polyethylene (Medpor) used for contour restoration and treatment. Br J Oral Maxillofac Surg 2000: 38: 44-9.

96. Ramirez OM. Mandibular matrix implant system: a method to restore skeletal support to the lower face. Plast Reconstr Surg 2000: 106: 176-89.

97. Yaremchuk MJ. Mandibular Augmentation. Plastic and Reconstructive Surgery 2000: 106: 697-706.

98. Fedok FG, van Kooten DW, Levin RJ. Temporal augmentation with a layered expanded polytetrafluoroethylene implant. Otolaryngol Head Neck Surg 1999: 120: 929-33.

99. Metzinger SE, McCollough EG, Campbell JP, Rousso DE. Malar Augmentation: A 5-Year Retrospective Review of the Silastic Midfacial Malar Implant. Archives of Otolaryngology–Head & Neck Surgery 1999: 125: 980-87.

100. Mendelsohn M, Dunlop G. Gore-tex augmentation grafting in rhinoplasty--is it safe? J Otolaryngol 1998: 27: 337-41.

101. Karras SC, Wolford LM. Augmentation genioplasty with hard tissue replacement implants. J Oral Maxillofac Surg 1998: 56: 549-52.

102. Yaremchuk MJ, Israeli D. Paranasal implants for correction of midface concavity. Plast Reconstr Surg 1998: 102: 1676-84; discussion 85.

103. Frodel JL, Lee S. The Use of High-Density Polyethylene Implants in Facial Deformities. Archives of Otolaryngology–Head & Neck Surgery 1998: 124: 1219-23.

104. Hirano S, Shoji K, Kojima H, Omori K. Use of hydroxyapatite for reconstruction after surgical removal of intraosseous hemangioma in the zygomatic bone. Plast Reconstr Surg 1997: 100: 86-90.

105. Abrahams JJ, Caceres C. Mandibular erosion from silastic implants: evaluation with a dental CT software program. AJNR Am J Neuroradiol 1998: 19: 519-22.

106. Semergidis TG, Migliore SA, Sotereanos GC. Alloplastic augmentation of the mandibular angle. J Oral Maxillofac Surg 1996: 54: 1417-23.

107. VUYK HD. Augmentation mentoplasty with solid silicone. Clinical Otolaryngology & Allied Sciences 1996: 21: 106-18.

108. Eppley BL, Sadove AM, Holmstrom H, Kahnberg KE. HTR polymer facial implants: a five-year clinical experience. Aesthetic Plast Surg 1995: 19: 445-50.

109. Matarasso A, Elias AC, Elias RL. Labial Incompetence: A Marker for Progressive Bone Resorption in Silastic Chin Augmentation. Plastic and Reconstructive Surgery 1996: 98: 1007-14.

110. Owsley TG, Taylor CO. The use of Gore-Tex for nasal augmentation: a retrospective analysis of 106 patients. Plast Reconstr Surg 1994: 94: 241-8; discussion 49-50.

111. Ono I, Gunji H, Suda K, Kaneko F, Yago K. Orbital reconstruction with hydroxyapatite ceramic implants. Scand J Plast Reconstr Surg Hand Surg 1994: 28: 193-8.

112. Blake GB, MacFarlane MR, Hinton JW. Titanium in reconstructive surgery of the skull and face. Br J Plast Surg 1990: 43: 528-35.

113. Moenning JE, Wolford LM. Chin augmentation with various alloplastic materials: a comparative study. Int J Adult Orthodon Orthognath Surg 1989: 4: 175-87.

114. Epker BN, Stella JP. Reconstruction of frontal and frontal-nasal deformities with prefabricated custom implants. J Oral Maxillofac Surg 1989: 47: 1272-6.

115. Pitanguy I, Martello L, Caldeira AML, Alexandrino A. Augmentation mentoplasty: A critical analysis. Aesthetic Plastic Surgery 1986: 10: 161-69.

116. Dann JJ, Epker BN. Proplast genioplasty: a retrospective study with treatment recommendations. Angle Orthod 1977: 47: 173-85.

117. Laub DR, Spohn W, Lash H, Weber J, Chase RA. Accurate reconstruction of traumatic bony contour defects of periorbital area with prefabricated silastic. J Trauma 1970: 10: 472-80.
